# Proteomics identifies complement protein signatures in patients with alcohol-associated hepatitis

**DOI:** 10.1172/jci.insight.174127

**Published:** 2024-05-08

**Authors:** Moyinoluwa Taiwo, Emily Huang, Vai Pathak, Annette Bellar, Nicole Welch, Jaividhya Dasarathy, David Streem, Craig J. McClain, Mack C. Mitchell, Bruce A. Barton, Gyongyi Szabo, Srinivasan Dasarathy, Alcohol Hepatitis Network (AlcHepNet) Consortium, Esperance A. Schaefer, Jay Luther, Le Z. Day, Xinshou Ouyang, Arumugam Suyavaran, Wajahat Z. Mehal, Jon M. Jacobs, Russell P. Goodman, Daniel M. Rotroff, Laura E. Nagy

**Affiliations:** 1 Department of Inflammation and Immunity,; 2 Department of Quantitative Health Sciences, and; 3 Department of Gastroenterology and Hepatology, Cleveland Clinic, Cleveland, Ohio, USA.; 4 Department of Family Medicine, Metro Health Medical Center, Cleveland, Ohio, USA.; 5 Department of Psychiatry and Psychology, Cleveland Clinic Lutheran Hospital, Cleveland, Ohio, USA.; 6 Department of Medicine, University of Louisville, Louisville, Kentucky, USA.; 7 Department of Internal Medicine, University of Texas Southwestern Medical Center, Dallas, Texas, USA.; 8 Department of Population and Quantitative Health Sciences, University of Massachusetts Medical School, Worcester, Massachusetts, USA.; 9 Department of Medicine, Beth Israel Deaconess Medical Center, Harvard Medical School, Boston, Massachusetts, USA.; 10 Department of Molecular Medicine, Case Western Reserve University, Cleveland, Ohio, USA.; 11 See Supplemental Acknowledgments for information on the AlcHepNet Consortium.; 12 Alcohol Liver Center, Division of Gastroenterology, Massachusetts General Hospital, Boston, Massachusetts, USA.; 13 Biological Sciences Division and Environmental Molecular Sciences Laboratory, Pacific Northwest National Laboratory, Richland, Washington, USA.; 14 Department of Internal Medicine, Yale School of Medicine, New Haven, Connecticut, USA.; 15 West Haven VA Medical Center, West Haven, Connecticut, USA,; 16 Endocrine Unit, Division of Gastroenterology, Massachusetts General Hospital, Boston, Massachusetts, USA.; 17 Endocrine and Metabolism Institute and; 18 Center for Quantitative Metabolic Research, Cleveland Clinic, Cleveland, Ohio, USA.

**Keywords:** Hepatology, Complement, Diagnostics, Hepatitis

## Abstract

Diagnostic challenges continue to impede development of effective therapies for successful management of alcohol-associated hepatitis (AH), creating an unmet need to identify noninvasive biomarkers for AH. In murine models, complement contributes to ethanol-induced liver injury. Therefore, we hypothesized that complement proteins could be rational diagnostic/prognostic biomarkers in AH. Here, we performed a comparative analysis of data derived from human hepatic and serum proteome to identify and characterize complement protein signatures in severe AH (sAH). The quantity of multiple complement proteins was perturbed in liver and serum proteome of patients with sAH. Multiple complement proteins differentiated patients with sAH from those with alcohol cirrhosis (AC) or alcohol use disorder (AUD) and healthy controls (HCs). Serum collectin 11 and C1q binding protein were strongly associated with sAH and exhibited good discriminatory performance among patients with sAH, AC, or AUD and HCs. Furthermore, complement component receptor 1-like protein was negatively associated with pro-inflammatory cytokines. Additionally, lower serum MBL associated serine protease 1 and coagulation factor II independently predicted 90-day mortality. In summary, meta-analysis of proteomic profiles from liver and circulation revealed complement protein signatures of sAH, highlighting a complex perturbation of complement and identifying potential diagnostic and prognostic biomarkers for patients with sAH.

## Abstract 2

Hepatic and circulating proteomic meta-analysis identifies complement protein signatures as well as potential diagnostic and prognostic biomarkers of alcohol-associated liver disease.

## Graphical abstract

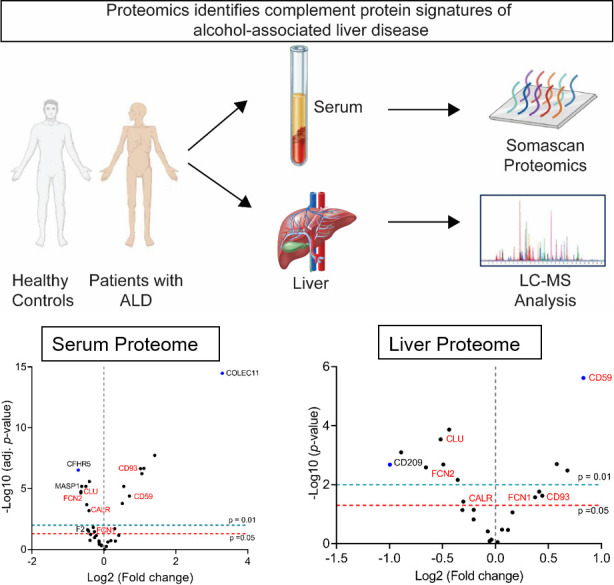

## Introduction

Alcohol-associated hepatitis (AH) is an acute, inflammatory clinical syndrome in active and chronic heavy drinkers and is characterized by rapid onset of jaundice and hepatic decompensation (1–3) with 28-day short-term mortality of up to 30%–40% from first presentation in severe cases (2), mortality increasing with concomitant bacterial infections, and multiorgan failure (4–6). Despite the progressive nature and high mortality rate of AH, no available medical therapies provide complete and sustained clinical benefit (7, 8). This is in part due to the complex and incompletely understood pathogenesis and diagnostic challenges associated with the disease. Corticosteroids, the recommended therapy for management of AH (9–11), have therapeutic value only in a subset of patients (8, 12). Due to the diagnostic challenges in patients with AH (13), including the risk, inconvenience, and cost associated with invasive liver biopsy, there is a strong need for the identification and development of biomarkers to effectively diagnose, manage, and predict clinical outcomes in patients with AH (14).

Complement, a system of over 50 circulating and membrane-bound proteins, is a vital part of the innate immune system involved in immune surveillance, clearance of cellular debris and pathogens, and tissue repair, thus contributing to maintaining homeostasis. However, if not properly regulated, it can contribute to uncontrolled inflammation. The complement system is activated by 1 or more of 3 pathways: the classical, lectin, or alternative pathway (AP) (15, 16).

Accumulating evidence from murine models indicates that complement activation and release of anaphylatoxins contributes to liver inflammation and drives progression of ethanol-induced liver injury (17, 18). For example, we and others have shown the involvement of complement in initiation and progression of hepatic inflammation and injury in response to chronic ethanol feeding in mice. Mice with a deficiency in C1q (19) or those treated with human C1 inhibitor, CINRYZE (20), were protected from chronic ethanol-induced liver injury. Further, inhibition of complement receptor 2-Crry–mediated activation of C3 decreased inflammatory responses and hepatic steatosis in ethanol-exposed mice (21). In contrast, complement factor D (CFD), a component of the AP, protects mice from chronic ethanol-induced injury (22).

Importantly, studies evaluating the impact of AH on the quantity and activation of circulating and hepatic complement provide insights into the involvement of complement in AH. Analysis of complement in liver explants from a small number of patients with AH (*n* = 3–5) revealed increased quantity of immunoreactive C1q, C3, C5, and C5aR (23). In a separate analysis of liver explants from 5 patients, expression of C1qR, C3aR, C5aR, and C5aR2 mRNA and C3b, iC3b, and C3c protein were also higher than in healthy controls (HCs) (22). In patients with AH enrolled by the Defeat Alcoholic Steatohepatitis (DASH) consortium, the concentration of both circulating C5a and CFBa are increased, while CFI and sC5b9 are decreased compared with HCs. Importantly, both CFI and sC5b9 are negatively associated with 90-day mortality in this cohort of patients with AH (24).

Given the dynamic dysregulation of complement in both murine models of alcohol-associated liver disease (ALD) and patients with AH, we hypothesized that the complement system may provide useful biomarkers in this disease process. In the current meta-analysis of circulating and hepatic proteome data from patients with severe AH (sAH), alcohol cirrhosis (AC), or alcohol use disorder (AUD) and HCs, we identified complement protein signatures of sAH with potential to serve as biomarkers for diagnostic and prognostic use in sAH.

## Results

### Identification of a complement proteome signature in patients with AH.

To identify complement proteome signatures in patients with AH, we used 2 independent protein expression data sets from liver (test cohort 1) and serum (test cohort 2) (25, 26). First, a list of complement proteins consisting of 80 soluble and membrane-associated proteins was compiled from literature (Supplemental Table 1; supplemental material available online with this article; https://doi.org/10.1172/jci.insight.174127DS1). From the compiled list, a total of 24 complement proteins (Supplemental Table 2) were found in the hepatic proteome data set; among these, 13 (7 decreased and 6 increased) were significantly different (Benjamini-Hochberg FDR-adjusted *P* value < 0.05) between patients with sAH and HCs (Table 1). The top 4 downregulated proteins are involved in the lectin and terminal pathway of complement activation: complement receptor (CD209; –1.99-fold), mannose-binding lectin 2 (MBL2; –1.85-fold), ficolin 2 (FCN2; –1.58-fold), and clusterin (CLU; –1.43-fold) (Table 1). The top 4 upregulated proteins are involved in the classical and terminal pathways: complement regulator, protectin (CD59; 1.78-fold), complement C1q subcomponent subunit A (C1qA; 1.60-fold) and subunit C (C1qC; 1.49-fold), and complement component C1q receptor 1 (C1qR1/CD93; 1.36-fold) (Table 1).

In the serum proteome data set, a total of 36 complement proteins were found from the total of 1,305 identified proteins, including the heterotrimers C8αβγ and C1qABC, heterodimer integrin α_2_/β_3_ (ITGα2/β3), and activated form of C4A (or C4Ab) (Supplemental Table 3). Of these, 23 complement proteins were significantly different (Benjamini-Hochberg FDR-adjusted *P* value < 0.05) across groups: sAH, AUD, and HCs (Table 2). These differences were predominantly between sAH and AUD, except for C1R, and between sAH and HCs, except for CFB, C9, C6, and ELANE. In contrast, only collectin-11 (COLEC11), C8αβγ, CFB, C9, and C1R were different between AUD and HCs (Table 2).

The top 4 downregulated serum proteins in sAH relative to HCs were complement factor H-related protein 5 (CFHR5; 0.48-fold), CLU (0.54-fold), MBL associated serine protease 1 (MASP1; 0.55-fold), and FCN2 (0.55) while the top 4 upregulated serum proteins in sAH relative to HCs were COLEC11 (15.94-fold), complement C7 (3.97-fold), decay-accelerating factor (CD55; 3.05-fold), and complement C1R (2.96-fold) (Table 2).

**Figure 1:**
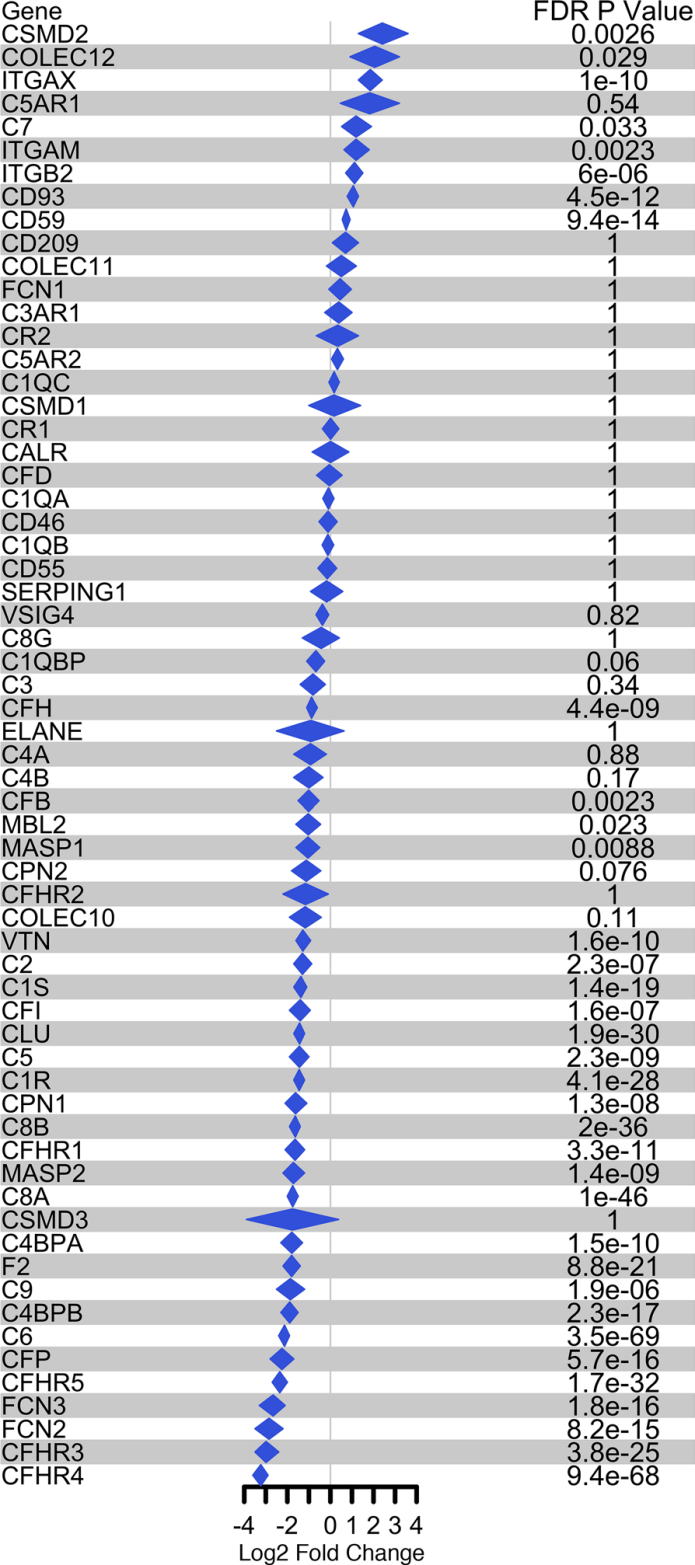
Complement protein plot of summarized log_2_ fold-change for sAH versus HC of 3 different bulk RNA-Seq data sets. Bonferroni FDR-adjusted *P* value < 0.05 is statistically significant.

Analysis of 3 publicly available bulk RNA-Seq data sets from studies comparing sAH with HC also revealed 63 complement and complement-associated genes. Among these genes, 35 (8 increased and 27 decreased) were differentially expressed (Bonferroni FDR-adjusted *P* value < 0.05) between AH and HC. CUB and Sushi multiple domains 2 (CSMD2; log fold-change [LogFC]: 2.41) and complement factor H-related protein 4 (CFHR4; LogFC: –3.23) were the top differentially expressed genes (DEGs) (Figure 1). Interestingly, C7 (LogFC: 1.19), FCN2 (LogFC: –2.85), and CFHR5 (LogFC: –2.36) genes were all among the top 5 DEGs (Figure 1), similar to the proteins affected by AH in both liver and serum (Tables 1 and 2).

### Overlap in the complement proteome signatures between liver and serum.

Thirteen complement proteins were identified in both liver and serum (Table 3). Eleven of these overlapping complement proteins were regulated in similar directions in sAH, with 8 down- and 3 upregulated, but the extent of up- and downregulation varied between liver and serum, except for FCN2, which was downregulated to 0.6-fold in both compartments (Table 3). Of note, 2 proteins, ficolin 1 (FCN1) and vitronectin (VTN), were differentially regulated between liver and serum, with both upregulated in the liver and downregulated in serum (Table 3). Further comparative analysis between the liver and serum complement proteome revealed that of these 11 overlapping proteins, only 5 were significantly different in both compartments between patients with sAH and HCs, including decreases in FCN2, CLU, and CALR and increases in CD59 and CD93 (C1qR1) (Table 3).

### Validation of select complement proteins.

**Figure 2:**
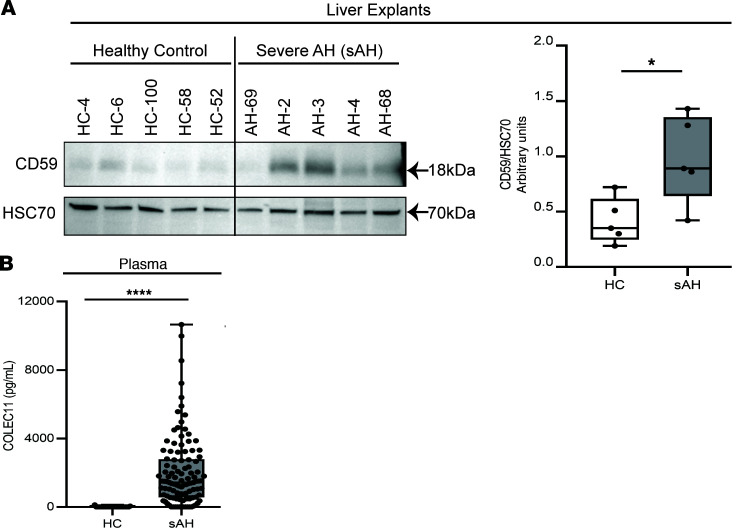
Validation of top upregulated complement proteins in liver and serum proteome of patients with sAH. (**A**) Western blot and ImageJ (NIH) quantification of CD59 expression in HC (*n* = 5) and sAH (*n* = 5) liver explants (validation cohort 1). Unpaired 2-tailed *t* test. (**B**) ELISA measurement of COLEC11 concentration in HCs (*n* = 23) and patients with sAH (*n* = 109) (validation cohort 2). Box-and-whisker plots show minimum and maximum values, lower and upper quartiles, and median, with each individual value represented as a point superimposed on the graph. Mann-Whitney *U* test. *P < 0.05, ****P < 0.0001.

Results from both liver and serum proteome data were verified by performing Western blot analysis and ELISA quantification of the complement proteins CD59 and COLEC11, respectively. These proteins were the top upregulated complement proteins in sAH relative to HC in the liver and serum proteomic data sets, respectively (Tables 1 and 2). In agreement with the liver proteome (Table 1), patients with sAH undergoing liver transplant showed increased expression of hepatic CD59 protein compared with HCs (validation cohort 1) (Figure 2A). Further, in our validation cohort 2, patients with sAH had elevated plasma COLEC11 protein concentration compared with HCs (Figure 2B), in agreement with the serum proteome (Table 2).

### Differentially expressed pathways in patients with sAH compared with HCs.

Using Reactome molecular pathway analysis (https://reactome.org/), we identified differentially expressed pathways connected to the complement proteins that were impacted by disease state. A total of 114 (57 up- and 57 downregulated; Supplemental Table 4) pathways were identified in the liver, with the top-regulated pathways being ER to Golgi Anterograde Transport, Membrane Trafficking, Coat Protein Complex II (COPII)-mediated vesicle transport, Cargo concentration in the ER; COPI-mediated anterograde transport, and Transport to the Golgi and subsequent modification. CD59 gene was seen to be common with these top-regulated pathways in the liver (Table 4).

A total of 177 (89 up- and 88 downregulated; Supplemental Table 5) pathways were identified in the serum. The top-regulated pathways are those involved in Binding and uptake of ligands by scavenger receptors, Scavenging by Class A Receptors, and Vesicle-mediated transport. Interestingly, MASP1, CALR, and COLEC11, all associated with the lectin pathway of complement activation, were common to these top-regulated pathways in the serum (Table 4).

### Circulating complement proteins correlate with clinical variables of interest.

Since hepatocytes are the main source of most of the circulating complement proteins (27), we sought to understand the relationship between these complement proteins and sAH by doing a correlation analysis. Spearman’s correlation analysis between complement proteins and clinical variables of interest showed that serum C1QBP, C8αβγ, F2, FCN1, ITGα2/β3, and MASP1 negatively correlated with Model for End-Stage Liver Disease (MELD) score, while CD55, CD59, CD93, CFD, CTSG, and FCN3 positively correlated with MELD (Supplemental Table 6).

Further, as albumin can be used as an indicator of the capacity of the liver to synthesize and secrete circulating proteins, we examined the correlation of complement proteins with albumin. Of the 18 patients with sAH, 10 (55.5%) had received albumin infusions at least once within the 14 days leading up to sample collection (Supplemental Table 7). However, there was no significant difference in the reported index albumin values between those who received albumin infusions and those who did not (Supplemental Figure 1). Of the 36 circulating complement proteins, only 4 correlated with albumin (Supplemental Table 6). Interestingly, of these, serum C1QBP and ITGα2/β3, which were negatively correlated, and CD93, which was positively correlated, are not secretory proteins. FCN1, which is a secreted protein, was negatively correlated with albumin. Taken together, these data indicate that changes in complement in sAH were not associated with decreased secretory capacity of the liver.

A point biserial correlation analysis between sAH and serum complement proteins showed that complement activators, C1QABC, C1R, C7, CFP, COLEC11; complement regulatory factors, CD55, CD59, CFH, SERPING1; complement receptor, CD93; and the complement-associated protein, CTSB, which cleaves complement C3, were positively correlated with sAH, and complement activators, C8αβγ, FCN2, MASP1; complement regulatory factors, CFI, CLU; and complement receptors, C1QBP, CALR, CFHR5, were negatively correlated (Supplemental Table 8).

### Serum complement proteins are associated with 90-day mortality in sAH.

**Figure 3:**
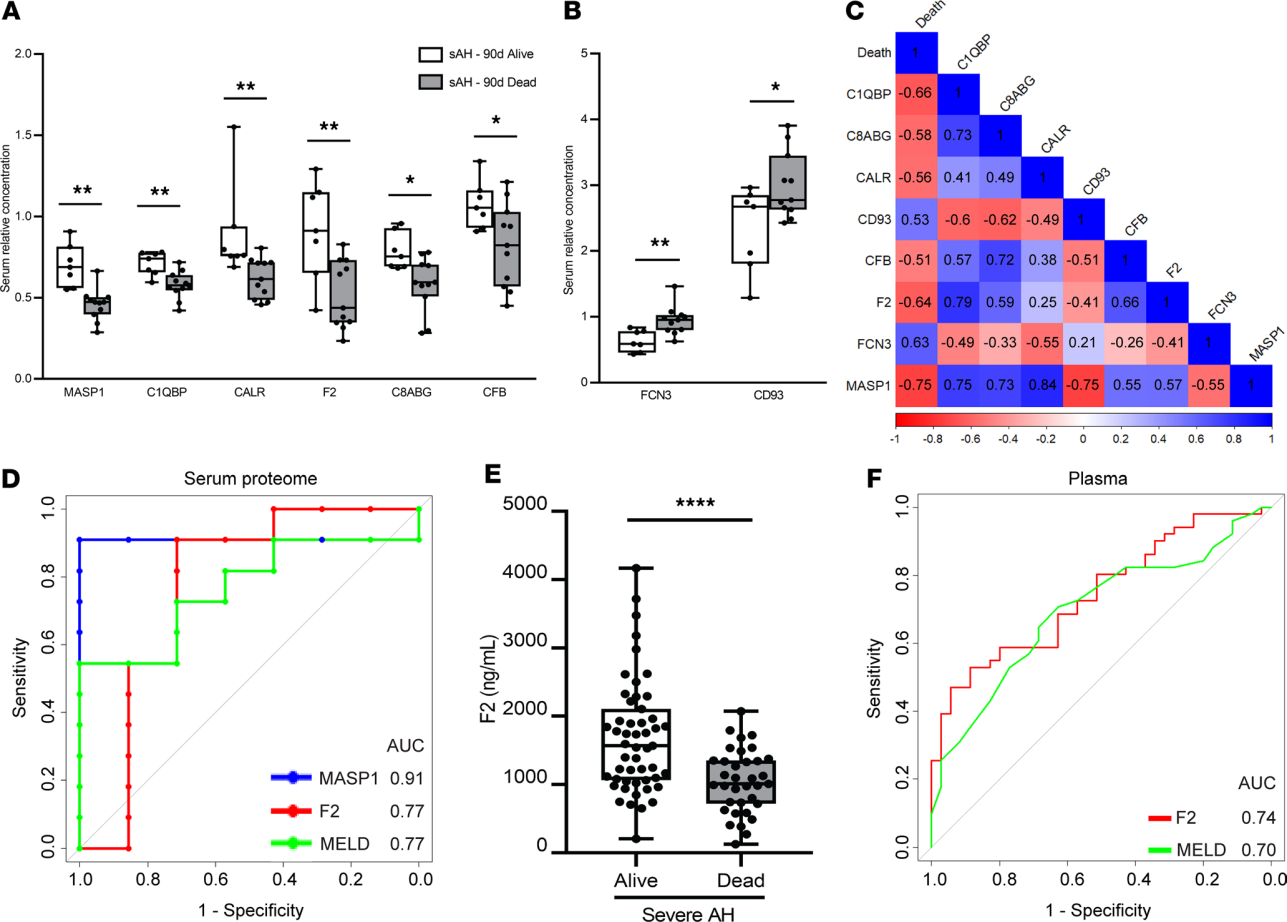
Circulating complement proteins are associated with 90-day mortality in patients with sAH. Serum relative concentration of complement proteins that (**A**) decreased and (**B**) increased in patients with sAH who died at 90 days. sAH-90d Alive, *n* = 7; sAH-90d Dead, *n* = 11 (test cohort 2). *P < 0.05, **P < 0.01 (Benjamini-Hochberg FDR-adjusted *P* value). (**C**) Point biserial correlation matrix of serum complement proteins associated with sAH 90-day mortality (Death). Correlation coefficients (*r*) are given within the blocks with blue and red blocks for positive and negative association, respectively. (**D**) Receiver operating characteristic (ROC) curves show area under curve (AUC) for MASP1 and F2, potential predictors of 90-day mortality, and MELD. (**E**) Plasma concentrations of F2 in alive (*n* = 51) and dead (*n* = 35) (validation cohort 3). Unpaired 2-tailed *t* test. ****P < 0.0001. (**F**) ROC curves show AUC for F2 and MELD. Box-and-whisker plots show minimum and maximum values, lower and upper quartiles, and median, with each individual value represented as a point superimposed on the graph.

In the cohort of patients (test cohort 2) used for the serum analysis, 11 (61%) of the 18 patients with sAH died within 90 days. Among all clinical parameters at diagnosis, only MELD was different between those dead and alive at 90 days (Supplemental Table 9). However, we found that 8 complement proteins had potential prognostic value. Patients who died had significantly lower relative serum concentrations of MASP1, C1QBP, CALR, F2, C8αβγ, and CFB (Figure 3A) and higher relative serum concentrations of FCN3 and CD93 (Figure 3B). All 8 of these complement proteins were significantly associated (Benjamini-Hochberg FDR-adjusted *P* value < 0.05) with 90-day mortality (Figure 3C). Next, we asked whether serum levels of these complement proteins could potentially predict 90-day mortality using multiple regression analysis. To select the suitable variables to be included in the regression model, a stepwise selection approach was used to identify possible predictors of 90-day mortality in patients with sAH from the following potential risk factor variables: age, sex, creatinine, albumin, MELD, and the 8 complement proteins (MASP1, C1QBP, CALR, F2, C8αβγ, CFB, FCN3, and CD93) with potential prognostic value. Results of this stepwise multivariate analysis identified complement proteins MASP1 and F2 as the only independent predictors of mortality; these proteins were therefore included in the regression model. None of the remaining variables outside the model met the entry criteria. Hosmer and Lemeshow test showed that there was no evidence of a lack of fit in the selected model (*P* = 0.7854).

Finally, the prognostic potential of MASP1 and F2 was assessed by plotting the receiver operating characteristic (ROC) curve following leave-one-out cross-validation (LOOCV). MASP1 gave an AUC of 0.91 (sensitivity 90.9% and specificity 100%) while F2 gave an AUC of 0.77 (sensitivity 90.9% and specificity 71.4%) (Figure 3D). Importantly, the AUC of MASP1 surpassed MELD’s prediction of 90-day mortality with AUC of 0.77 (sensitivity 54.5% and specificity 100%) (Figure 3D).

To validate this result, we measured circulating levels of MASP1 and F2 by ELISA in validation cohort 3, comparing patients with sAH who were alive with those who died during a follow-up period of 90 days. While our initial serum proteome findings revealed that deceased patients had significantly lower relative serum concentrations of both MASP1 and F2 (Figure 3A), our validation results yielded some nuanced differences. Notably, plasma concentrations of MASP1 were not significantly different between patients who were alive or deceased (Supplemental Figure 2A). In contrast, for F2, plasma concentrations were significantly lower in deceased patients (Figure 3E), consistent with the results from the serum proteome data set (Figure 3A). Furthermore, the AUC value of 0.74 from our validation cohort (Figure 3F) closely mirrors the AUC of 0.77 from the serum proteome analysis (Figure 3D), and is higher than AUC of 0.70 for MELD.

### Multiple complement proteins discriminate sAH from HC, AUD, and AC.

**Figure 4:**
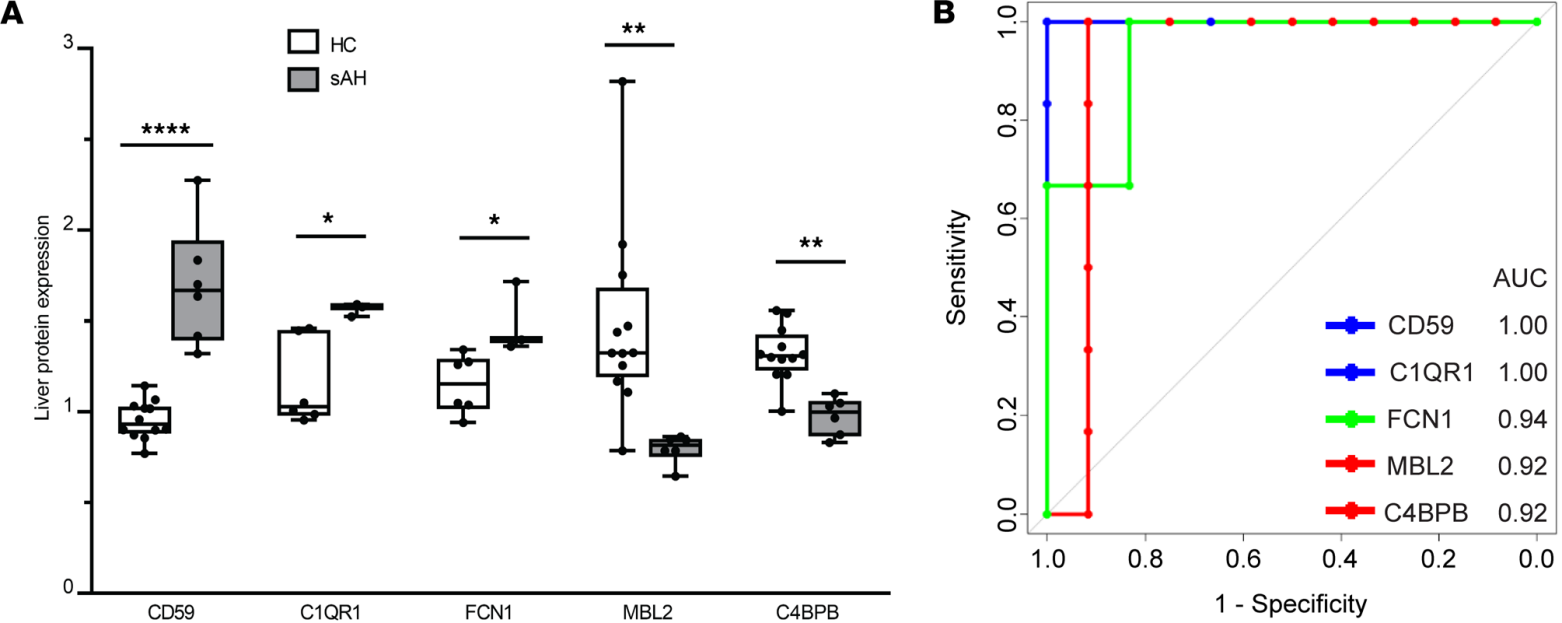
Top discriminatory complement proteins in liver proteome of patients with sAH. (**A**) Liver protein expression of the top 5 discriminatory complement proteins in HCs and patients with sAH (test cohort 1). Box-and-whisker plots show minimum and maximum values, lower and upper quartiles, and median, with each individual value represented as a point superimposed on the graph (*n* = 3–12). *P < 0.05, **P < 0.01, ****P < 0.0001 (Benjamini-Hochberg FDR-adjusted *P* value). (**B**) ROC curves show AUC of the top 5 discriminatory complement proteins distinguishing patients with sAH from HCs.

**Figure 5:**
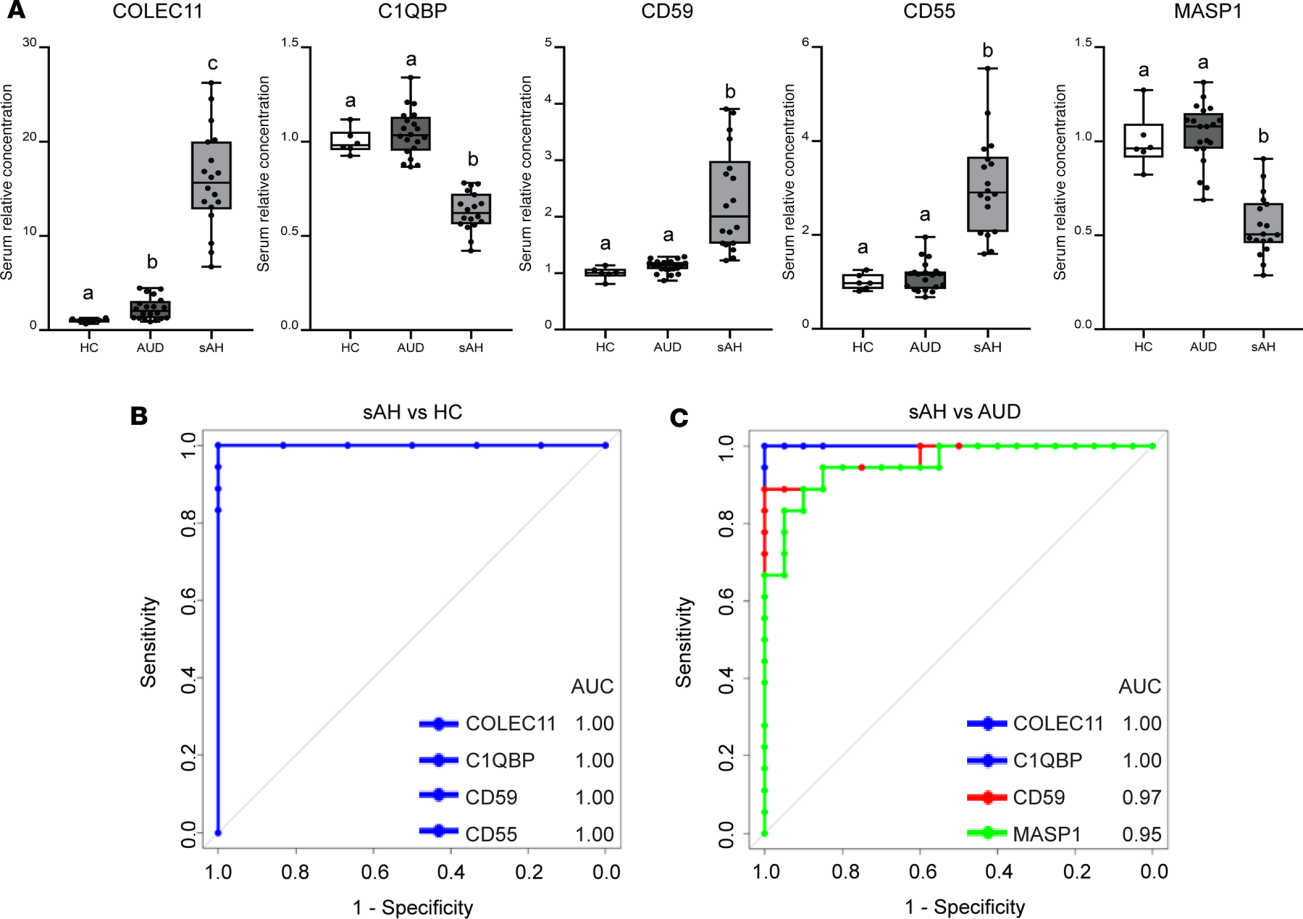
Complement protein dysregulation in serum proteome of patients with AUD and sAH (test cohort 2). (**A**) Serum relative concentration of select complement proteins in HC (*n* = 6), AUD (*n* = 20), and sAH (*n* = 18). Box-and-whisker plots show minimum and maximum values, lower and upper quartiles, and median, with each individual value represented as a point superimposed on the graph. Values with different superscripts are significantly different, *P* < 0.05. One-way ANOVA.ROC curves show AUC distinguishing patients with sAH from (**B**) HCs and (**C**) patients with AUD.

We investigated whether liver and serum complement proteins could discriminate sAH from HC, AUD, and AC by analyzing ROC curves for these proteins. Thirteen hepatic complement proteins discriminated sAH from HCs with AUC values ranging 0.76–1.00 (Supplemental Table 10). The top 5 discriminatory complement proteins were CD59 (AUC: 1.00; sensitivity 100% and specificity 100%), C1QR1 (AUC: 1.00; sensitivity 100% and specificity 100%), FCN1 (AUC: 0.94; sensitivity 100% and specificity 83.3%), MBL2 (AUC: 0.92; sensitivity 100% and specificity 91.7%), and C4BPB (AUC: 0.92; sensitivity 100% and specificity 91.7%) (Figure 4). Also, multiple serum complement proteins discriminated sAH from either HCs or patients with AUD with no clinical evidence of AH (Supplemental Table 11). COLEC11 and C1QBP discriminated sAH from HCs and AUD, both with AUC curves of 1.00 (sensitivity 100% and specificity 100%) and 1.00 (sensitivity 100% and specificity 100%) (Figure 5).

**Figure 6:**
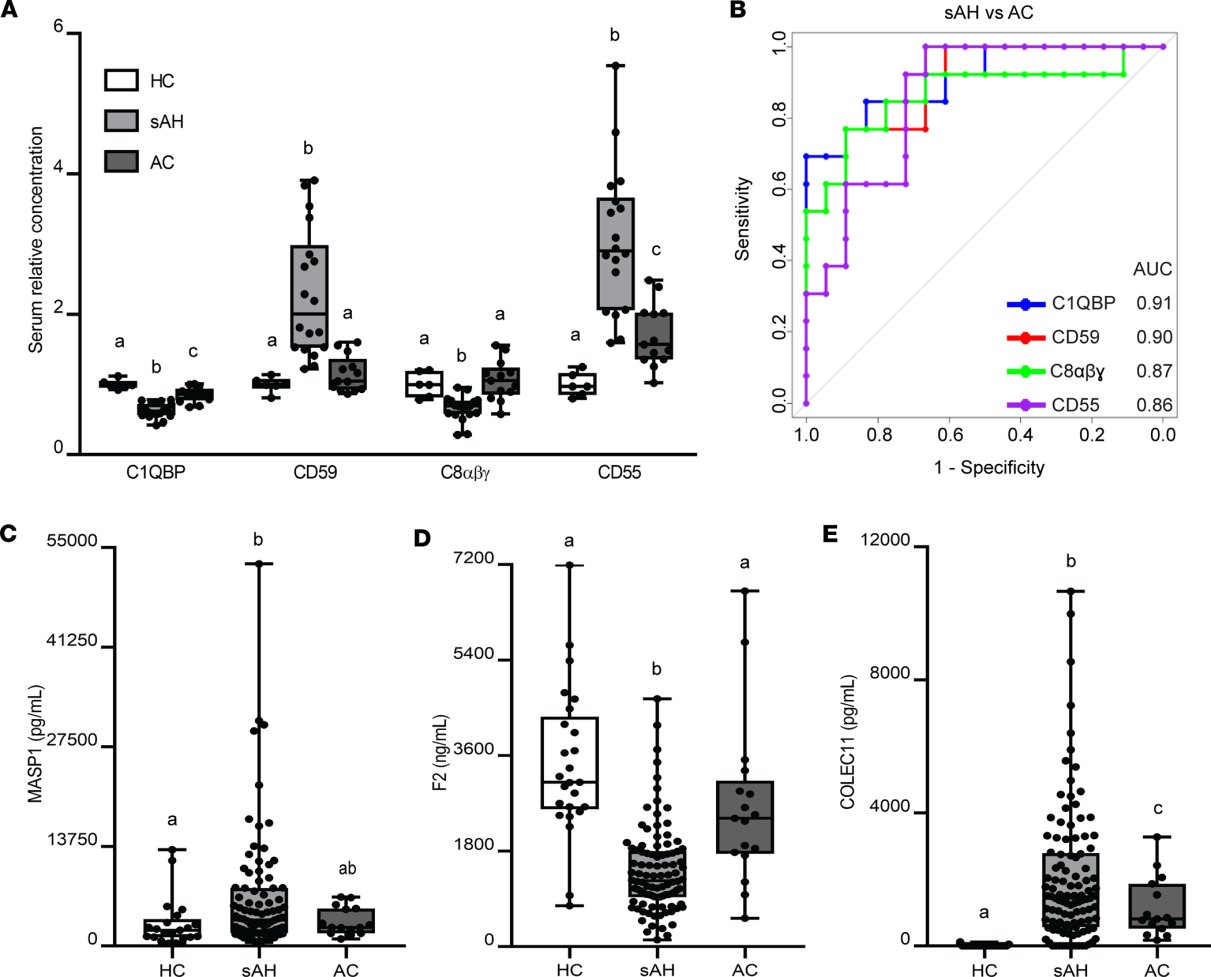
Complement protein changes from HC in patients with sAH and AC. (**A**) Serum relative concentration in HC (*n* = 6), sAH (*n* = 18), and AC (*n* = 13) (test cohort 2). (**B**) ROC curves show AUC of the top 4 discriminatory serum complement proteins distinguishing patients with sAH from AC. (**C**–**E**) MASP1, F2, and COLEC11 plasma concentrations, respectively, in HC (*n* = 21–25), sAH (*n* = 91–109), and AC (*n* = 15–17) (validation cohorts 2 and 3). Box-and-whisker plots show minimum and maximum values, lower and upper quartiles, and median, with each individual value represented as a point superimposed on the graph. Values with different superscripts are significantly different, *P* < 0.05. One-way ANOVA.

Importantly, we sought to compare the complement proteome signature between patients with sAH and AC, given the complexities involved in distinguishing these 2 stages of ALD in the setting of chronic liver disease. First, we compared complement proteins in compensated versus decompensated AC; no significant differences (Benjamini-Hochberg FDR-adjusted *P* value < 0.05) were seen between these 2 groups of AC (Supplemental Table 12). Because no differences were seen between compensated and decompensated AC and because of the limited sample size, we combined both groups to investigate the potential of serum complement proteins as discriminative markers between sAH and AC. Our results showed that 17 complement proteins exhibited significant differences (Benjamini-Hochberg FDR-adjusted *P* value < 0.05) between these 2 groups (Table 5). The diagnostic potential of these proteins was further substantiated by AUC values ranging from 0.69 to 0.91. Notably, C1QBP, CD59, C8αβγ, and CD55 emerged as top discriminatory markers, demonstrating both high sensitivity and specificity (Figure 6, A and B, and Table 5).

Next, in our validation cohort 3, we measured circulating concentrations of MASP1, F2, and COLEC11 by ELISA in patients with sAH or AC and HC. For MASP1, in contrast with the higher relative concentrations in AC compared with sAH in the serum proteome (Table 5), plasma concentrations in the validation cohort were not significantly different between patients with AC compared to sAH or HC (Figure 6C). However, plasma concentrations of F2 were significantly lower in sAH compared with both HC and AC (Figure 6D). Further, plasma concentrations of COLEC11 in sAH were significantly higher compared with both HC and AC (Figure 6E), with a 1.8-fold elevation compared with AC, consistent with the differences observed in the serum proteome (Table 5).

### Dysregulation of complement proteome dysregulation correlates with pro-inflammatory cytokines in AH.

To explore a potential mechanism by which dysregulated complement could drive the progression of AH, we delved into the interplay between complement proteins and inflammatory cytokines using a matched, independent data set encompassing proteomics and multiplex analysis from patients with sAH (test cohort 3). We identified 45 complement proteins from proteomics and 17 pro-inflammatory cytokines (inclusive of both classical and nonclassical markers of inflammation) from the multiplex data. Spearman’s correlation analysis revealed both significantly positive and negative associations between complement proteins and pro-inflammatory cytokines (Supplemental Table 14). Notably, IFN-α significantly correlated with 11 complement proteins. Additionally, the chemokines CXCL1 and CX3CL1, along with interleukins IL-17E and IL-18, each correlated with 4 complement proteins (Supplemental Table 14). A particularly interesting finding was the negative correlation of complement component receptor 1-like protein (CR1L), a negative regulator of complement activation, with 7 pro-inflammatory cytokines (IL-1α, IL-1β, IL-8, IL-17C, IL-18, TNF-α, and CCL20) (Supplemental Table 14).

## Discussion

In this meta-analysis, we sought to identify complement protein signatures in patients with ALD. Complement protein signatures were manually identified by comparing hepatic and serum proteomic profiles of patients with ALD. Expression of multiple components of complement activation pathways, as well as receptors, proteases, regulators, and complement-associated proteins, was perturbed in both liver and serum of patients with ALD. Analysis of RNA-Seq data from livers of patients with sAH and HCs also revealed that multiple components of the complement system were perturbed in patients with sAH. These data provide insights into the pathophysiology of ALD and identify potential therapeutic targets. Importantly, serum complement signatures demonstrated a strong discriminatory ability to distinguish patients with sAH from individuals with AC and AUD and HCs. Furthermore, these signatures were associated with and predictive of 90-day mortality in patients with sAH.

One of the important findings of our proteomic analysis was identification of perturbations in classical and lectin, but not alternative, complement activation pathways. In the classical pathway, multiple complement-related proteins involved in the precise regulation of this pathway were impacted by sAH in both the liver and serum. C1R and C1S, serine proteases usually found in complex with inactive circulating C1q (the C1 complex), are required to initiate and complete the classical pathway activation (28, 29). Here, hepatic and serum C1R and hepatic C1S (*P* = 0.09) were increased in patients with sAH. Binding of C1q to its receptor mediates several cellular processes, including phagocytic uptake of apoptotic cells and pathogens; interaction of C1q with its receptors and binding proteins can either enhance or inhibit its function (30, 31). Expression of C1q, as well as multiple C1q receptors and C1q binding proteins, was detected in liver and serum and was differentially impacted by sAH. For example, interaction between C1q and C1qR1, also called CD93, a type I membrane glycoprotein, enhances phagocytic function (32). Expression of CD93 was elevated in both liver and serum of patients with sAH compared with HCs. In contrast, the C1q binding proteins, C1QBP and CALR, limit binding of C1q to immune complexes, thus inhibiting the classical pathway of complement activation (33, 34). The interaction of C1QBP and C1q also inhibits complement hemolytic activity (34). Here, both these inhibitory C1q binding proteins were lower in the serum of patients with sAH, and hepatic expression of CALR was lower in patients with sAH. Another C1q binding protein, CD209, a C-type lectin receptor, also inhibits the classical pathway via interactions with the IgG binding site of C1q (35). Expression of CD209 was decreased in liver of patients with sAH. Collectively, increased expression of C1R and C1S, involved in activation of the classical pathway, in parallel with decreased expression of multiple C1q binding proteins that inhibit activation of the classical pathway, suggest that patients with sAH may have enhanced classical pathway activity, while increased expression of CD93 may provide enhanced phagocytic function in sAH.

In the lectin pathway, multiple components were also perturbed by sAH in both liver and serum. The ficolins (FCN1, FCN2, and FCN3) and the collectins (MBL, COLEC10, and COLEC11) initiate activation of the lectin pathway while forming complexes with MASPs (36–39). Here, hepatic and serum FCN2, FCN3, MBL2, and MASP1 were decreased, while hepatic FCN1 was higher in patients with sAH. Also, while serum FCN1 decreased, COLEC11 increased in patients with sAH. Our validation cohort also showed increased plasma COLEC11 concentrations in patients with sAH compared with HCs; very low to undetectable concentrations were found in HCs, consistent with previous reports (39, 40). Complex perturbations in the expression of lectin components make it difficult to determine the impact of AH on this pathway. However, pathway analysis of both the liver and serum proteome suggested that the lectin pathway was activated.

This study also focused on complement regulators, which are vital for sustaining the balance between complement activation and control in order to protect cells and tissues from unwanted inflammation and complement-mediated damage (41, 42). For example, SERPING1 (C1INH) regulates the classical pathway by binding and inactivating C1R and C1S proteases, leading to C1 complex dissociation (43, 44). C4BP negatively regulates classical and lectin pathways by preventing formation of C3 convertase and facilitating its dissociation (45, 46). Also, CD55 inhibits both classical pathway and AP by inhibiting the formation of new C3- and C5-convertases and accelerating their dissociation (47, 48). Additionally, CFI inhibits all 3 pathways by cleaving C3b and C4b while requiring the presence of other several cofactor proteins including C4BP to function maximally (49, 50). CLU, CD59, and VTN are regulators preventing MAC assembly; while CLU and VTN bind to C5b-7, CD59 binds to C5b-8 or C9 (28). Here, these regulators were differentially impacted by sAH. Serum SERPING1 and CD55 were increased while VTN (*P* = 0.1) and CFI were decreased in patients with sAH compared with HCs, consistent with our previous studies where plasma CFI was decreased in both moderate AH and sAH compared with HC (24). Hepatic C4BPA and C4BPB were decreased while hepatic and serum CLU were decreased and CD59 was increased in patients with sAH. Our validation cohort also showed increased expression of hepatic CD59 in patients with sAH compared with HCs. Pathway analysis revealed CD59 to be associated with vesicle trafficking pathways in both liver and serum proteome, consistent with previous reports where CD59 aids vesicle signaling in pancreatic β cells (51). Collectively, decreased expression of CFI coupled with decreased expression of the C4BPs would result in increased C3 cleavage, potentiating complement activation, while increased CD59 expression would likely protect damaged or dying liver cells from complement-mediated cell lysis.

The diagnosis and prognosis of AH in chronic liver disease present major challenges (13, 14), particularly due to the similarities in clinical and laboratory findings in patients with sAH and AC. This complexity underscores the need for developing noninvasive biomarkers for AH. Liver biopsy, not commonly advocated for suspected AH cases, rarely changes the clinical diagnosis, except when atypical clinical features are present, further underscoring the importance of noninvasive markers. In our study, we identified multiple hepatic and serum complement proteins with potential diagnostic abilities to distinguish patients with sAH from those with AC or AUD and HCs. Generally, there were few significant differences in complement proteins between AUD and HC groups, suggesting that heavy drinking alone does not lead to complement dysregulation. Of particular efficacy, COLEC11 in both serum proteome and plasma validation was able to discriminate between sAH and AC. COLEC11 was low to undetectable in plasma of HCs but was up to 265- and 2-fold higher in patients with sAH and AC, respectively. Thus, determining circulating levels of COLEC11 may offer valuable diagnostic insights for AH.

Importantly, MASP1 and F2 independently predicted 90-day mortality in patients with sAH, with MASP1 outperforming MELD in predictive accuracy, while F2 was just as good as MELD. MASP1 also performed better than the previously reported concordance statistic (equivalent to AUC) for MELD of 0.86 (52). Despite some differences observed in the mortality prediction of MASP1 between serum proteome and plasma, F2 showed consistent predictive ability in both data sets. Considering the liver’s crucial role in synthesizing coagulation factors like F2, our study’s finding of altered F2 in patients with sAH aligns with expectations. Notably, F2 emerged as a significant predictor (*P* < 0.0001) of mortality in these patients. This is consistent with prior research indicating reduced thrombin production in chronic liver disease, worsening with disease progression (53). Moreover, studies have associated coagulation abnormalities with increased morbidity and mortality in chronic liver disease (54). In our study, the perturbed levels of F2, particularly the significantly lower concentrations in deceased patients at 90 days, underscore its prognostic value in AH.

Delving into the interplay between complement dysregulation and inflammation, we found that CR1L, a negative regulator of complement activation, was negatively associated with pro-inflammatory cytokines in sAH. Its established role in regulating complement-dependent cytotoxicity is consistent with an involvement in restraining excessive inflammation. The negative correlation with key inflammatory cytokines, notably those involved in acute and chronic inflammatory responses and liver diseases, like IL-1β, IL-18, and TNF-α (55), reinforces the hypothesis that CR1L might have a protective role in AH. This protective mechanism could be crucial in mitigating liver damage and progression of AH, highlighting the potential therapeutic value of targeting CR1L in managing AH-related inflammation. However, further research is needed to elucidate the exact mechanisms by which CR1L interacts with these cytokines and to explore its potential as a therapeutic target in AH.

The strengths of this meta-analysis study include the pooled estimate of effect from multiple data sets, which may reduce the probability of false negative results, and the use of MELD score in both test and validation cohorts to categorize the severity of AH. The major limitation is that data from other metabolic dysfunction-associated liver diseases were not included. For instance, some of the complement proteins identified in this study, including C1QBP, C4BPA, CLU, and SERPING1, have also been identified in proteomic analyses as potential biomarkers in metabolic dysfunction-associated steatotic liver disease/metabolic dysfunction-associated steatohepatitis (56–58). However, to the best of our knowledge, no studies to date have reported COLEC11 to be involved in any chronic liver disease. Thus, comparing complement proteome between alcohol- and metabolic-associated liver diseases would provide valuable insights as to the diagnostic specificity of complement proteins in liver diseases. Other limitations are the heterogeneity of study demographics, methods utilized, and data quality of test cohorts. Integration of additional proteomic studies would provide more robust results and help in identifying potentially novel biomarkers for use in ALD.

In summary, this study provides insights into complement protein signatures and the complex, dynamic perturbation of the complement system in ALD. We show, to the best of our knowledge, for the first time the involvement of the lectin pathway of complement activation in AH. Circulating COLEC11 was positively and MASP1 negatively associated with sAH, both with good discriminatory performance distinguishing sAH from those with AC and AUD and HCs. Interestingly, CR1L was negatively associated with pro-inflammatory cytokines, adding another layer of understanding to the complex interplay of immune responses in ALD. Additionally, circulating MASP1 not only correlated with but also predicted 90-day mortality in patients with sAH. While these proteins show potential for being developed as biomarkers, further studies are still needed to elucidate the involvement and contribution of classical and lectin pathway of complement activation in ALD.

## Methods

### Human participants

#### Sex as a biological variable.

Both sexes were included in all human cohorts.

#### Test cohort 1: liver proteomics data.

The hepatic proteomics data were generated by liquid chromatography-mass spectrometry (LC-MS/MS) analysis of deidentified liver samples acquired by University of Louisville (U of L) and Johns Hopkins University (JHU) Clinical Resources Center for Alcoholic Hepatitis Investigations (R24AA025017). Samples used included HCs (*n* = 12; U of L 7 and JHU 5) and patients with sAH (*n* = 6 JHU) with an average MELD score of 37.2 ± 1.8. The data set was generated from the MS resources at Pacific Northwest National Laboratory (accession number MSV000089168; https://massive.ucsd.edu/ProteoSAFe/static/massive.jsp) reported by Hardesty et al. 2022 (25). Demographic and clinical parameters of this study cohort have been reported (25).

#### Test cohort 2: serum proteomics data.

The circulating proteomic data were acquired using the aptamer-based, proteomic SomaScan platform by Luther et al. (26). Serum samples from HCs (*n* = 6); patients with AUD (*n* = 20) (AUD diagnosed based on guidelines presented in the *Diagnostic and Statistical Manual of Mental Disorders*, fifth edition) as reported by Luther et al. (26); patients with mild (*n* = 21) (MELD < 10), moderate (*n* = 15) (MELD 11–20), or severe (*n* = 18) (MELD > 20) AH; and patients with AC (*n* = 13) were included in the cohort. However, in the current study, we focused on 4 groups (HCs, AUD, sAH, and AC). Clinical characteristics of the study population have been previously reported by Luther et al. (26)

#### Test cohort 3: serum proteomics and multiplex data.

Multiplex data from a Luminex assay matched to proteomics data from plasma samples of patients with sAH (*n* = 10) were used. These patients were recruited into the observational arm of the AlcHepNet (NCT03850899). Plasma samples for proteomics were processed using Pierce Top 12 (T12) Abundant Protein Depletion Spin Columns (Thermo Fisher Scientific 85164). Depleted samples were then analyzed using LC-MS/MS, and the resultant data were processed with Progenesis QI and the Mascot search engine. The subset of proteins with a Mascot score greater than 30, with an emphasis on identifying complement proteins, were analyzed;.Utilizing both the multiplex and proteomics data, we conducted Spearman’s correlation analysis to understand the association between the normalized abundance values of these complement proteins and the concentrations of pro-inflammatory cytokines.

### Validation cohorts: Western blot and ELISA

Additional cohorts of patients were used as validation cohorts for Western blots and ELISAs. Demographic and clinical characteristics of all validation cohorts are provided in Supplemental Table 13.

#### Validation cohort 1.

Immunoblotting of liver tissue was conducted to validate expression of CD59. Samples were obtained from explanted livers in patients with sAH (*n* = 5) and wedge biopsies from healthy donors (*n* = 5) during liver transplantation from the Clinical Resource for Alcoholic Hepatitis Investigations at JHU.

#### Validation cohort 2.

COLEC11 was measured by ELISA in 134 participants: 24 HCs and 110 patients with sAH. HCs together with their clinical and demographic data were obtained from the Northern Ohio Alcohol Center (NOAC) biorepository (ClinicalTrials.gov NCT03224949). Patients with a clinical diagnosis of sAH (MELD score ≥ 20) at admission were recruited as part of the DASH consortium, a multicenter (Cleveland Clinic, University of Texas Southwestern, University of Massachusetts, and U of L), randomized, double-blind controlled trial (59) (NCT01809132 and NCT03224949). Details of patient recruitment, as well as the inclusion and exclusion criteria for the DASH consortium, have been previously reported by Dasarathy et al. (59).

#### Validation cohort 3.

MASP1, F2, and COLEC11 were measured in 115 participants: 98 and 17 patients with sAH and AC, respectively. Samples from patients with AC together with their clinical and demographic data were obtained from the NOAC biorepository. Samples from patients with sAH together with their clinical and demographic data were obtained from the AlcHepNet observational study biorepository (NCT03850899).

### RNA-Seq analysis

Raw counts were obtained from 3 different bulk RNA-Seq data sets: Massey et al. 2021 (11 HC and 10 AH from liver explant tissue) (60), JHU (7 HC and 13 AH from liver explant tissue) (61), and Argemi et al. 2019 (divided into 2 subsets: 10 HC and 11 AH liver explant tissue and 10 HC and 18 AH nonexplant liver tissue) (62). The R package DESeq2 was used to analyze the log_2_ fold-change for AH versus HC for each of the 4 comparisons (3 data sets of explants and the 1 nonexplant). Next, a meta-analysis was performed across the separate RNA-Seq studies using the metafor R package (63). Summarized log_2_ fold-change values and a meta-analyzed *P* value were obtained using a random effects model with original study log_2_ fold-change values. The summaries for each individual complement protein gene were then plotted using the forestplot package in R.

### Liver homogenates and immunoblotting

Frozen liver tissue from human participants was homogenized in lysis buffer, and protein concentration was measured using the DC Protein Assay (Bio-Rad). Samples were denatured at 37°C in Laemmli buffer for 15 minutes. Samples were separated on 8%–16% SDS-PAGE gels (Bio-Rad), transferred to PVDF membranes with a wet transfer apparatus (Bio-Rad), and blocked in 5% milk in TBS-Tween. Membrane was probed with antibody specific for CD59 (Cell Signaling Technology; 65055), and HSC70 (Santa Cruz Biotechnology; sc-7298) was used as loading control. Membrane was developed using Immobilon Western developing reagents (MilliporeSigma). Chemiluminescence was visualized using iBright FL1500 imaging system (Thermo Fisher Scientific). Arbitrary density of immune-positive bands was quantified using ImageJ software.

### Sample collection and ELISA measurement

Blood samples were obtained within 48 hours of patient enrollment. Plasma was then separated, aliquoted, and stored at –80°C until use. Aliquoted samples were thawed on ice prior to measurement. For COLEC11 (catalog MBS2883570) and MASP1 (catalog MBS2507077), samples were diluted 20-fold in sample diluent provided by the manufacturer, while for F2 (catalog MBS2019898), samples were diluted 2,000-fold in PBS. All assays were performed according to manufacturer’s instruction (MyBioSource).

### Reactome analysis

Molecular pathway analysis was conducted with the analysis tools of Reactome, version 83 (https://reactome.org/). Pathway identifier mapping, overrepresentation, and expression analysis were merged using the analysis tools. The Pathway Analysis with Down-weighting of Overlapping Genes method was used as the gene set analysis method. This method computes a gene set score by taking the weighted average of the absolute values of moderated gene *t* scores (64). The Reactome analysis generated the following parameters: log FC (log_2_ fold-change for AH over HC), AveExpr (average expression in log_2_ counts per million reads, across all samples), *t* (moderated *t* statistic from the test that the logFC differs from 0), *P* value (raw *P* value from the test that the logFC differs from 0), adj. *P*. Val (Benjamini-Hochberg FDR-adjusted *P* value), and B (log odds that the gene is differentially expressed). An FDR *P* value < 0.05 was used to determine pathways considered to be significantly overrepresented.

### Statistics

Continuous variables are presented as means ± SEM. Two-group comparison of continuous variables was done either by unpaired 2-tailed *t* test or Mann-Whitney *U* test based on the results of Shapiro-Wilk normality test (except when stated otherwise). For multiple-group comparison of continuous variables, 1-way ANOVA using the general linear models (GLM) procedure was done. Data were log-transformed as necessary to obtain a normal distribution, with follow-up comparisons done by least square means testing.

Categorical variables are presented as counts and percentages with Fisher’s exact tests used for comparison. Correlation analysis was used to assess the association of complement proteins, continuous clinical variables, sAH, 90-day mortality, and pro-inflammatory cytokines. While Spearman’s correlation was used between complement proteins and clinical variables, as well as between these proteins and pro-inflammatory cytokines, point biserial correlation was used for the association between complement proteins and sAH as well as between these proteins and 90-day mortality. Correlation coefficient (*r*) was used to measure the strength and direction of association and *P* value to determine the significance of the correlation coefficient. ROC curves were generated to evaluate the diagnostic and 90-day mortality prognostic potentials of complement proteins in AH. Only the complement proteins that were significantly different between groups were used for ROC analysis. To limit overfitting, the LOOCV method was used while fitting in a general linear regression model. To select the suitable variables to establish the prognostic model, the stepwise multiple regression selection approach was used. A significance level of 0.3 (SLENTRY = 0.3) and 0.35 (SLSTAY = 0.35) is required to allow a variable into the model and to stay in the regression model, respectively. SAS (SAS Enterprise Guide 8.2) was used for stepwise regression analysis and 1-way ANOVA using the GLM procedure. The R package Corrplot was used to generate the correlation matrix while the package pROC was used to generate the ROC plots. Youden’s index from the LOOCV procedure was used to assess the performance of the complement proteins; AUC, optimal threshold, and corresponding sensitivity and specificity values are reported. All other statistical tests, as well as volcano plots and box-and-whisker plots, used GraphPad Prism 9. Box-and-whisker plots show each individual value as a point superimposed on the graph, minimum and maximum values, and lower and upper quartile, as well as median. An FDR approach was used to control for multiple testing, and Benjamini-Hochberg FDR-adjusted *P* values < 0.05 were considered statistically significant except where otherwise stated.

### Study approval

All studies were approved by the Institutional Review Boards of all participating institutions, and all study participants consented in writing prior to data and sample collection.

### Data availability

Data are available in the Supporting Data Values Excel file.

## Author contributions

MT and LEN conceptualized the work. DASH consortium (GS, AB, NW, JD, DS, SD, CJM, MCM, BAB), AlcHepNet Consortium, JL, and RPG collected the samples. JL, EAS, RPG, LZD, and JMJ provided proteomics data sets. WZM, XO, and AS provided proteomics and Luminex data sets. MT and EH performed experimental validation. MT, VP, DMR, and LEN analyzed the data. MT wrote first draft of the manuscript. MT and LEN wrote and edited the manuscript. All authors reviewed and approved the manuscript.

## Supplementary Material

Supplemental data

Unedited blot and gel images

Supplemental table 14

Supporting data values

## Figures and Tables

**Table 1:**
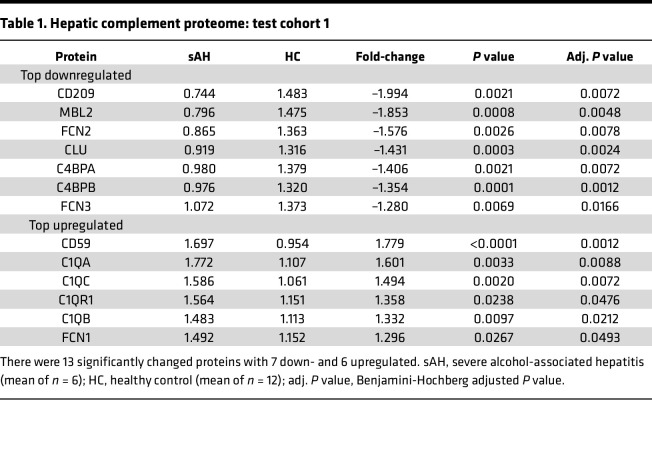
Hepatic complement proteome: test cohort 1

**Table 2:**
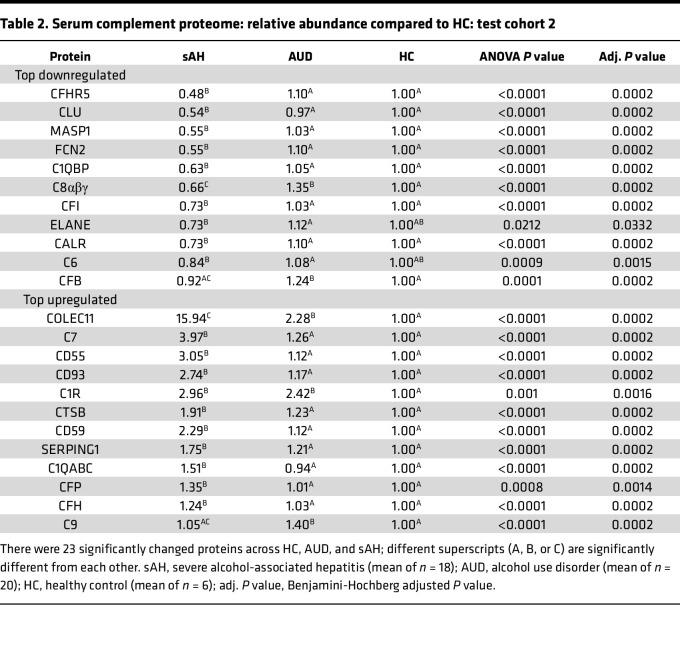
Serum complement proteome: relative abundance compared to HC: test cohort 2

**Table 3:**
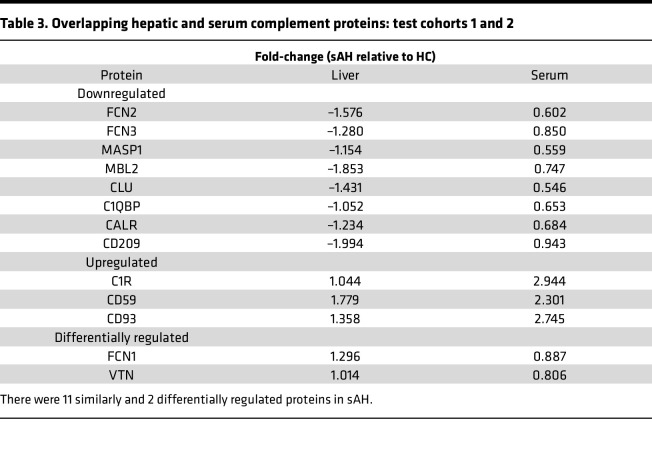
Overlapping hepatic and serum complement proteins: test cohorts 1 and 2

**Table 4:**
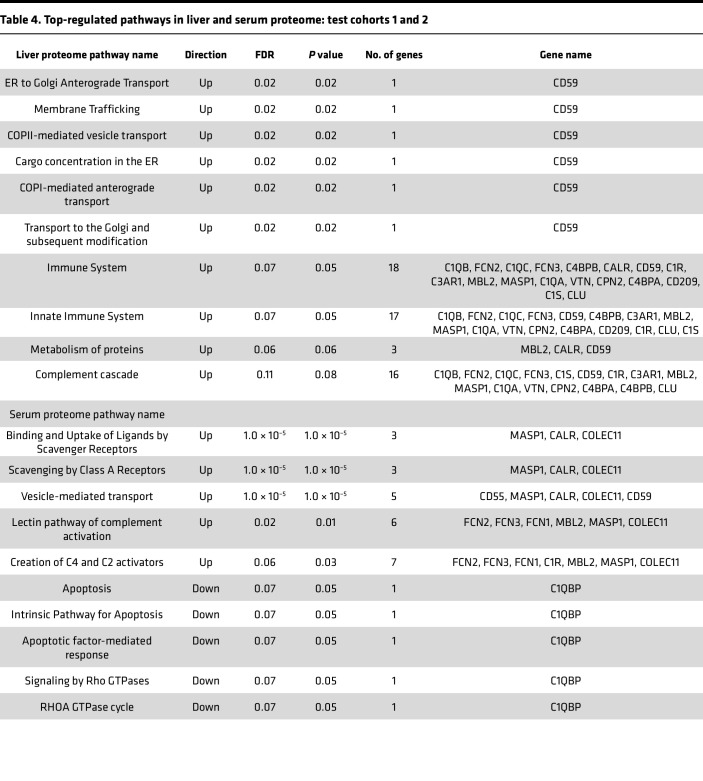
Top-regulated pathways in liver and serum proteome: test cohorts 1 and 2

**Table 5:**
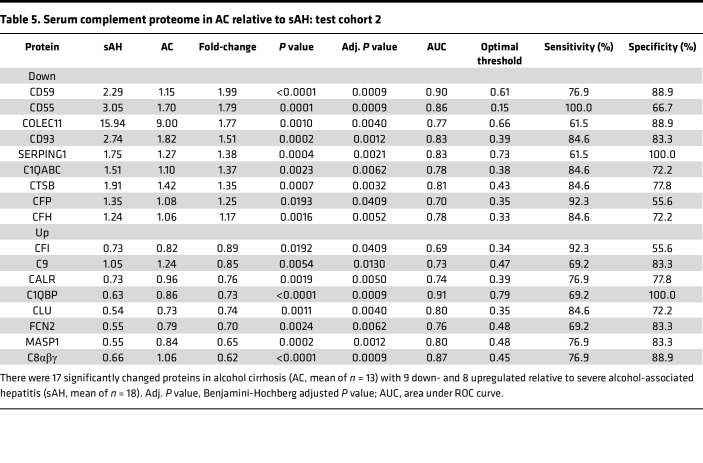
Serum complement proteome in AC relative to sAH: test cohort 2

## Version 1 04/04/2024

In-Press Preview

## Version 2 05/08/2024

Electronic publication

## Version 3 10/07/2024

author affiliation update
