# Supplementary material for: Proteomics identifies complement protein signatures in patients with alcohol-associated hepatitis
Source: JCI Insight. 2024 May 8;9(9):e174127. doi: 10.1172/jci.insight.174127 (PMC11141929; doi:10.1172/jci.insight.174127)
Supplement: Supplemental data [file jciinsight-9-174127-s012.pdf]

Complement Protein Signatures in Patients with Alcohol-Associated Hepatitis

Moyinoluwa Taiwo, Emily Huang, Vai Pathak, Annette Bellar, Nicole Welch, Jaividhya Dasarathy, David Streem, Craig J McClain, Mack Mitchell, Bruce Barton, Gyongyi Szabo, Srinivasan Dasarathy, AlcHepNet Consortium, Esperance A. Schaefer, Jay Luther, Le Z. Day, Arumugam Suyavaran, Wajahat Z. Mehal, Jon Jacobs, Russell P. Goodman, Daniel M. Rotroff, Laura E. Nagy

CONTENTS

SUPPLEMENTARY TABLES 1

Supplementary Table 1. Complement and complement-associated genes 1

Supplementary Table 2. Complement proteins in liver proteome: Test cohort 1 3

Supplementary Table 3. Complement proteins in serum proteome: Test cohort 2 4

Supplementary Table 4. Liver pathways 6

Supplementary Table 5. Serum pathways 8

Supplementary Table 6. Spearman's correlation of serum complement proteins with MELD & albumin in patients with sAH: significant findings only 10

Supplementary Table 7. Albumin infusion status in patients with severe AH 11

Supplementary Table 8. Point-biserial correlation analysis between serum complement proteins and severe AH: significant findings only 12

Supplementary Table 9. Clinical features of patients with sAH (serum proteome - Test cohort 2) 13

Supplementary Table 10. LOOCV ROC curve analysis of complement proteins significantly changed in liver proteome 14

Supplementary Table 11. LOOCV ROC curve analysis of serum complement proteins with significant changes: only proteins with AUC ≥ 0.70 included 15

Supplementary Table 12. Serum complement proteins in compensated vs decompensated AC. 17

Supplementary Table 13. Clinical features of patients used in the validation cohorts 18

Supplementary Figure 1 20

Supplementary Figure 2 21

Supplemental Acknowledgments: Alcohol Hepatitis Network Consortium 22

SUPPLEMENTARY TABLES

Supplementary Table 1. Complement and complement-associated genes

Component	Symbol	Component	Symbol
Complement C1q subcomponent subunit A	C1QA	Collectin-10	COLEC10
Complement C1q subcomponent subunit B	C1QB	Collectin-11	COLEC11
Complement C1q subcomponent subunit C	C1QC	Collectin-12	COLEC12
Complement C1r subcomponent	C1R	Plasma protease C1 inhibitor	SERPING1
Complement C1s subcomponent	C1S	C4b-binding protein alpha chain	C4BPA
Complement C2	C2	C4b-binding protein beta chain	C4BPB
Complement C2A	C2A	Carboxypeptidase N catalytic chain	CPN1
Complement C2B	C2B	Carboxypeptidase N subunit 2	CPN2
Complement C3	C3	Clusterin	CLU
Complement C3A	C3A	Complement factor H	CFH
Complement C3a-desArg	C3adesArg	Complement factor I	CFI
Complement C3B	C3B	Complement decay-accelerating factor	CD55
Complement iC3B	iC3B	Membrane cofactor protein	CD46
Complement C3C	C3C	Protectin	CD59
Complement C3D	C3D	Vitronectin	VTN
Complement C4	C4	CUB and sushi domain-containing protein 1	CSMD1
Complement C4-A	C4A	CUB and sushi domain-containing protein 2	CSMD2
Complement C4-B	C4B	CUB and sushi domain-containing protein 3	CSMD3
Complement C5	C5	Neutrophil elastase	ELANE
Complement C5A	C5A	Prothrombin	F2
Complement C5B	C5B	Complement receptor type 1	CR1
Complement component C6	C6	Complement receptor type 2	CR2
Complement component C7	C7	Integrin alpha-M	ITGAM
Complement C8	C8	Integrin alpha-X	ITGAX
Complement C8 alpha chain	C8A	V-set and immunoglobulin domain-containing protein 4	VSIG4
Complement C8 beta chain	C8B	Complement factor H-related protein 1	CFHR1
Complement C8 gamma chain	C8G	Complement factor H-related protein 2	CFHR2

Complement component C9	C9	Complement factor H-related protein 3	CFHR3
Complement factor B	CFB	Complement factor H-related protein 4	CFHR4
Complement factor D	CFD	Complement factor H-related protein 5	CFHR5
Properdin	CFP		CRIT
Ficolin-1	FCN1	C3a anaphylatoxin chemotactic receptor	C3AR1
Ficolin-2	FCN2	C5a anaphylatoxin chemotactic receptor 1	C5AR1
Ficolin-3	FCN3	C5a anaphylatoxin chemotactic receptor 2	C5AR2
Mannose-binding lectin 1	MBL1	Calreticulin	CALR
Mannose-binding lectin 2	MBL2	Complement component 1 Q subcomponent-binding protein	C1QBP
Mannan-binding lectin serine protease 1	MASP1	Complement component C1q receptor	CD93
Mannan-binding lectin serine protease 2	MASP2	Cluster of Differentiation 209	CD209
Mannan-binding lectin serine protease 3	MASP3	Integrin beta-2	ITGB2
Complement component receptor 1-like protein	CRIL	Integrin alpha-2/beta-3	Integrin α2/β3

Supplementary Table 2. Complement proteins in liver proteome: Test cohort 1

**Protein**	**sAH**	**HC**	**Fold change**	***p.* value**	**adj. *p. value***
C1QA	1.772	1.107	1.601	0.0033	0.0088
C1QB	1.483	1.113	1.332	0.0097	0.0212
C1QC	1.586	1.061	1.494	0.0020	0.0072
C1R	1.278	1.224	1.044	0.3355	0.4110
C1S	1.307	1.168	1.118	0.0870	0.1228
FCN1	1.492	1.152	1.296	0.0267	0.0493
FCN2	0.865	1.363	-1.576	0.0026	0.0078
FCN3	1.072	1.373	-1.280	0.0069	0.0166
MASP1	1.051	1.213	-1.154	0.1512	0.2016
MBL2	0.796	1.475	-1.853	0.0008	0.0048
C4BPA	0.980	1.379	-1.406	0.0021	0.0072
C4BPB	0.976	1.320	-1.354	0.0001	0.0012
CD59	1.697	0.954	1.779	<0.0001	0.0012
CLU	0.919	1.316	-1.431	0.0003	0.0024
CPN2	1.077	1.242	-1.153	0.0712	0.1082
VTNC	1.213	1.196	1.014	0.8876	0.8876
C1QBP	1.167	1.227	-1.052	0.3853	0.4403
C1QR1	1.564	1.151	1.358	0.0238	0.0476
C3AR	1.107	1.137	-1.027	0.7305	0.7969
C5AR1	1.310	1.207	1.086	0.3425	0.4110
CALR	1.029	1.270	-1.234	0.0376	0.0645
CALR3	1.000	1.241	-1.242	0.0721	0.1082
CD209	0.744	1.483	-1.994	0.0021	0.0072
VSIG4	1.179	1.224	-1.039	0.8201	0.8558

24 complement proteins. sAH, severe alcohol- associated hepatitis (mean of n=6); HC, Healthy control (mean of n=12). adj. *p*. value, Benjamini-Hochberg adj. *p*. value

Supplementary Table 3. Complement proteins in serum proteome: Test cohort 2

Protein	sAH	AUD	HC	FC sAH/HC	FC sAH/AUD	Anova *p*. value	adj. *p. value*
C1QBP	0.63[b]	1.05[a]	1.00[a]	0.630	0.602	<0.0001	0.0002
COLEC11	15.94[c]	2.28[b]	1.00[a]	15.943	6.982	<0.0001	0.0002
C7	3.97[b]	1.26[a]	1.00[a]	3.966	3.150	<0.0001	0.0002
CD59	2.29[b]	1.12[a]	1.00[a]	2.289	2.047	<0.0001	0.0002
CD55	3.05[b]	1.12[a]	1.00[a]	3.050	2.721	<0.0001	0.0002
FCN2	0.55[b]	1.10[a]	1.00[a]	0.552	0.501	<0.0001	0.0002
CD93	2.74[b]	1.17[a]	1.00[a]	2.736	2.340	<0.0001	0.0002
MASP1	0.55[b]	1.03[a]	1.00[a]	0.551	0.533	<0.0001	0.0002
CFI	0.73[b]	1.03[a]	1.00[a]	0.725	0.707	<0.0001	0.0002
C8αβγ	0.66[c]	1.35[b]	1.00[a]	0.660	0.488	<0.0001	0.0002
CLU	0.54[b]	0.97[a]	1.00[a]	0.538	0.557	<0.0001	0.0002
CFB	0.92[ac]	1.24[b]	1.00[a]	0.920	0.742	0.0001	0.0002
CTSB	1.91[b]	1.23[a]	1.00[a]	1.906	1.555	<0.0001	0.0002
C1QABC	1.51[b]	0.94[a]	1.00[a]	1.506	1.606	<0.0001	0.0002
CFHR5	0.48[b]	1.10[a]	1.00[a]	0.481	0.439	<0.0001	0.0002
CALR	0.73[b]	1.10[a]	1.00[a]	0.727	0.659	<0.0001	0.0002
CFH	1.24[b]	1.03[a]	1.00[a]	1.242	1.210	<0.0001	0.0002
CFP	1.35[b]	1.01[a]	1.00[a]	1.347	1.327	0.0008	0.0014
SERPING1	1.75[b]	1.21[a]	1.00[a]	1.753	1.452	<0.0001	0.0002
C9	1.05[ac]	1.40[b]	1.00[a]	1.047	0.749	<0.0001	0.0002
C1R	2.96[b]	2.42[b]	1.00[a]	2.961	1.222	0.001	0.0016
C6	0.84[b]	1.08[a]	1.00[ab]	0.841	0.776	0.0009	0.0015
FCN3	0.82[a]	0.89[a]	1.00[a]	0.824	0.927	0.269	0.3026
CTSG	1.63[a]	1.25[a]	1.00[a]	1.630	1.302	0.1611	0.1933
C5	0.99[a]	1.01[a]	1.00[a]	0.990	0.982	0.9461	0.9461
MBL2	0.84[b]	1.05[a]	1.00[ab]	0.844	0.803	0.1107	0.1485
CD209	0.96[a]	0.83[a]	1.00[a]	0.962	1.165	0.2255	0.2619
CFD	0.99[a]	1.08[a]	1.00[a]	0.992	0.915	0.3572	0.3782
ELANE	0.73[b]	1.12[a]	1.00[ab]	0.726	0.647	0.0212	0.0332
VTN	0.77[a]	0.98[a]	1.00[a]	0.772	0.791	0.1114	0.1485
C2	1.05[a]	1.10[a]	1.00[a]	1.049	0.952	0.437	0.4495
ITGα2/β3	1.76[b]	1.94[b]	1.00[a]	1.758	0.906	0.0629	0.0944
FCN1	0.89[a]	0.98[a]	1.00[a]	0.885	0.899	0.1492	0.1852
C4AB	1.44[b]	0.68[a]	1.00[ab]	1.442	2.110	0.0754	0.1086
C3	1.21[a]	1.19[a]	1.00[a]	1.211	1.022	0.3333	0.3636

F2	0.67[a]	0.91[a]	1.00[a]	0.671	0.740	0.1395	0.1794

36 complement proteins, different superscript (a, b, or c) are significantly different from each other. sAH, severe alcohol-associated hepatitis (mean of n=18); AUD, Alcohol-use Disorder (mean of n=20); HC, Healthy control (mean of n=6); FC, Fold Change; adj. p. value, Benjamini-Hochberg adj. p. value

Supplementary Table 4. Liver pathways

Name	Direction	FDR	Pvalue	Number of Genes	MeanAbsT0	MeanWeightT0	av_foldchange	sig.
ER to Golgi Anterograde Transport	Up	0.016	0.016	1	2.380165238	2.149554794	0.68740412	FALSE
Membrane Trafficking	Up	0.016	0.016	1	2.380165238	2.149554794	0.68740412	FALSE
COPII-mediated vesicle transport	Up	0.016	0.016	1	2.380165238	2.149554794	0.68740412	FALSE
Cargo concentration in the ER	Up	0.016	0.016	1	2.380165238	2.149554794	0.68740412	FALSE
COPI-mediated anterograde transport	Up	0.016	0.016	1	2.380165238	2.149554794	0.68740412	FALSE
Transport to the Golgi and subsequent modification	Up	0.016	0.016	1	2.380165238	2.149554794	0.68740412	FALSE
Immune System	Up	0.073	0.048	17	0.644151321	0.650375403	0.018101364	FALSE
Innate Immune System	Up	0.067	0.051	16	0.89493431	0.917605355	0.026228384	FALSE
Metabolism of proteins	Up	0.064	0.062	3	1.043372842	0.411358107	0.036577299	FALSE
Complement cascade	Up	0.109	0.077	15	0.810748492	0.804565802	0.063516209	FALSE
Asparagine N-linked glycosylation	Up	0.068	0.084	2	1.029782186	0.82073874	0.287736582	FALSE
Post-translational protein modification	Up	0.068	0.084	2	1.029782186	0.82073874	0.287736582	FALSE
Initial triggering of complement	Up	0.161	0.133	8	1.043664338	0.986083381	0.091613084	FALSE
Creation of C4 and C2 activators	Up	0.161	0.133	8	1.043664338	0.986083381	0.091613084	FALSE
Regulation of Complement cascade	Up	0.156	0.145	12	0.837608085	1.009384096	0.158521947	FALSE
Neutrophil degranulation	Up	0.154	0.149	3	0.939900669	0.837111288	0.180322904	FALSE
Classical antibody-mediated complement activation	Up	0.175	0.158	5	1.213501854	1.417490049	0.336484979	FALSE
C-type lectin receptors (CLRs)	Down	0.291	0.272	1	0.43972183	0.554351468	-0.533088995	FALSE
CD209 (DC-SIGN) signaling	Down	0.291	0.272	1	0.43972183	0.554351468	-0.533088995	FALSE
Butyrophilin (BTN) family interactions	Down	0.291	0.272	1	0.43972183	0.554351468	-0.533088995	FALSE
Platelet degranulation	Down	0.322	0.304	1	0.198973292	0.238826758	-0.250991815	FALSE
Terminal pathway of complement	Down	0.322	0.304	1	0.198973292	0.238826758	-0.250991815	FALSE
Antimicrobial peptides	Down	0.322	0.304	1	0.198973292	0.238826758	-0.250991815	FALSE
Platelet activation, signaling and aggregation	Down	0.322	0.304	1	0.198973292	0.238826758	-0.250991815	FALSE
Response to elevated platelet cytosolic Ca2+	Down	0.322	0.304	1	0.198973292	0.238826758	-0.250991815	FALSE
Cell-Cell communication	Down	0.254	0.308	1	0.35084284	-0.448206716	-0.465741267	FALSE
Toll Like Receptor 4 (TLR4) Cascade	Down	0.254	0.308	1	0.35084284	-0.448206716	-0.465741267	FALSE
Toll Like Receptor TLR1:TLR2 Cascade	Down	0.254	0.308	1	0.35084284	-0.448206716	-0.465741267	FALSE
Toll-like Receptor Cascades	Down	0.254	0.308	1	0.35084284	-0.448206716	-0.465741267	FALSE
Toll Like Receptor 2 (TLR2) Cascade	Down	0.254	0.308	1	0.35084284	-0.448206716	-0.465741267	FALSE
Immunoregulatory interactions between a Lymphoid and a non-Lymphoid cell	Down	0.254	0.308	1	0.35084284	-0.448206716	-0.465741267	FALSE
Signal regulatory protein family interactions	Down	0.254	0.308	1	0.35084284	-0.448206716	-0.465741267	FALSE
Diseases of metabolism	Down	0.254	0.308	1	0.35084284	-0.448206716	-0.465741267	FALSE
Surfactant metabolism	Down	0.254	0.308	1	0.35084284	-0.448206716	-0.465741267	FALSE
Regulation of TLR by endogenous ligand	Down	0.254	0.308	1	0.35084284	-0.448206716	-0.465741267	FALSE
Diseases associated with surfactant metabolism	Down	0.254	0.308	1	0.35084284	-0.448206716	-0.465741267	FALSE
Defective CSF2RB causes SMDP5	Down	0.254	0.308	1	0.35084284	-0.448206716	-0.465741267	FALSE
Defective CSF2RA causes SMDP4	Down	0.254	0.308	1	0.35084284	-0.448206716	-0.465741267	FALSE
SARS-CoV-1 Infection	Down	0.254	0.308	1	0.35084284	-0.448206716	-0.465741267	FALSE
SARS-CoV-1-host interactions	Down	0.254	0.308	1	0.35084284	-0.448206716	-0.465741267	FALSE
SARS-CoV-1 activates/modulates innate immune responses	Down	0.254	0.308	1	0.35084284	-0.448206716	-0.465741267	FALSE
Generic Transcription Pathway	Down	0.349	0.333	1	0.193910328	0.233870255	-0.227773114	FALSE
RNA Polymerase II Transcription	Down	0.349	0.333	1	0.193910328	0.233870255	-0.227773114	FALSE
Gene expression (Transcription)	Down	0.349	0.333	1	0.193910328	0.233870255	-0.227773114	FALSE
RUNX1 and FOXP3 control the development of regulatory T lymphocytes (Tregs)	Down	0.349	0.333	1	0.193910328	0.233870255	-0.227773114	FALSE
Transcriptional regulation by RUNX1	Down	0.349	0.333	1	0.193910328	0.233870255	-0.227773114	FALSE
Vesicle-mediated transport	Up	0.32	0.363	3	0.095720393	-0.045522312	0.183451971	FALSE
Adaptive Immune System	Down	0.435	0.458	3	-0.076942682	-0.509632963	-0.37025374	FALSE
Metabolism	Up	0.558	0.536	1	-0.553102028	-0.468366043	0.170390342	FALSE
Acyl chain remodelling of PC	Up	0.558	0.536	1	-0.553102028	-0.468366043	0.170390342	FALSE
Acyl chain remodelling of PS	Up	0.558	0.536	1	-0.553102028	-0.468366043	0.170390342	FALSE
Acyl chain remodelling of PE	Up	0.558	0.536	1	-0.553102028	-0.468366043	0.170390342	FALSE
Glycerophospholipid biosynthesis	Up	0.558	0.536	1	-0.553102028	-0.468366043	0.170390342	FALSE
Phospholipid metabolism	Up	0.558	0.536	1	-0.553102028	-0.468366043	0.170390342	FALSE
Metabolism of lipids	Up	0.558	0.536	1	-0.553102028	-0.468366043	0.170390342	FALSE
Extracellular matrix organization	Up	0.555	0.553	1	-0.645231376	-0.596753908	0.152680262	FALSE
Elastic fibre formation	Up	0.555	0.553	1	-0.645231376	-0.596753908	0.152680262	FALSE
Molecules associated with elastic fibres	Up	0.555	0.553	1	-0.645231376	-0.596753908	0.152680262	FALSE
Integrin cell surface interactions	Up	0.555	0.553	1	-0.645231376	-0.596753908	0.152680262	FALSE
Syndecan interactions	Up	0.555	0.553	1	-0.645231376	-0.596753908	0.152680262	FALSE
Non-integrin membrane-ECM interactions	Up	0.555	0.553	1	-0.645231376	-0.596753908	0.152680262	FALSE
ECM proteoglycans	Up	0.555	0.553	1	-0.645231376	-0.596753908	0.152680262	FALSE
Lectin pathway of complement activation	Down	0.617	0.603	4	-0.526041732	-0.81004297	-0.243659369	FALSE
Hemostasis	Down	0.616	0.624	2	-0.631046864	-0.578082549	-0.093065389	FALSE
Signaling by GPCR	Up	0.615	0.635	1	-0.946119852	-0.933505766	0.081337706	FALSE
Class A/1 (Rhodopsin-like receptors)	Up	0.615	0.635	1	-0.946119852	-0.933505766	0.081337706	FALSE
Peptide ligand-binding receptors	Up	0.615	0.635	1	-0.946119852	-0.933505766	0.081337706	FALSE
GPCR downstream signalling	Up	0.615	0.635	1	-0.946119852	-0.933505766	0.081337706	FALSE
G alpha (i) signalling events	Up	0.615	0.635	1	-0.946119852	-0.933505766	0.081337706	FALSE
GPCR ligand binding	Up	0.615	0.635	1	-0.946119852	-0.933505766	0.081337706	FALSE
Leishmania infection	Up	0.615	0.635	1	-0.946119852	-0.933505766	0.081337706	FALSE
Purinergic signaling in leishmaniasis infection	Up	0.615	0.635	1	-0.946119852	-0.933505766	0.081337706	FALSE
Cell recruitment (pro-inflammatory response)	Up	0.615	0.635	1	-0.946119852	-0.933505766	0.081337706	FALSE
Parasitic Infection Pathways	Up	0.615	0.635	1	-0.946119852	-0.933505766	0.081337706	FALSE
Ficolins bind to repetitive carbohydrate structures on the target cell surface	Down	0.65	0.654	3	-0.809979879	-0.676584785	-0.169632069	FALSE
SARS-CoV Infections	Down	0.688	0.667	2	-0.664465775	-1.193421966	-0.245429259	FALSE
SARS-CoV-2 Infection	Down	0.688	0.667	2	-0.664465775	-1.193421966	-0.245429259	FALSE
SARS-CoV-2 activates/modulates innate and adaptive immune responses	Down	0.688	0.667	2	-0.664465775	-1.193421966	-0.245429259	FALSE
SARS-CoV-2-host interactions	Down	0.688	0.667	2	-0.664465775	-1.193421966	-0.245429259	FALSE
ER-Phagosome pathway	Down	0.721	0.747	1	-0.923833304	-0.988854937	-0.111930957	FALSE
Antigen processing-Cross presentation	Down	0.721	0.747	1	-0.923833304	-0.988854937	-0.111930957	FALSE
Influenza Infection	Down	0.721	0.747	1	-0.923833304	-0.988854937	-0.111930957	FALSE
Virus Assembly and Release	Down	0.721	0.747	1	-0.923833304	-0.988854937	-0.111930957	FALSE
Assembly of Viral Components at the Budding Site	Down	0.721	0.747	1	-0.923833304	-0.988854937	-0.111930957	FALSE
Cellular responses to stress	Down	0.721	0.747	1	-0.923833304	-0.988854937	-0.111930957	FALSE
Scavenging by Class F Receptors	Down	0.721	0.747	1	-0.923833304	-0.988854937	-0.111930957	FALSE
ATF6 (ATF6-alpha) activates chaperones	Down	0.721	0.747	1	-0.923833304	-0.988854937	-0.111930957	FALSE
Unfolded Protein Response (UPR)	Down	0.721	0.747	1	-0.923833304	-0.988854937	-0.111930957	FALSE
ATF6 (ATF6-alpha) activates chaperone genes	Down	0.721	0.747	1	-0.923833304	-0.988854937	-0.111930957	FALSE
N-glycan trimming in the ER and Calnexin/Calreticulin cycle	Down	0.721	0.747	1	-0.923833304	-0.988854937	-0.111930957	FALSE
Cellular responses to stimuli	Down	0.721	0.747	1	-0.923833304	-0.988854937	-0.111930957	FALSE
Calnexin/calreticulin cycle	Down	0.721	0.747	1	-0.923833304	-0.988854937	-0.111930957	FALSE
Class I MHC mediated antigen processing & presentation	Down	0.721	0.747	1	-0.923833304	-0.988854937	-0.111930957	FALSE
Antigen Presentation: Folding, assembly and peptide loading of class I MHC	Down	0.721	0.747	1	-0.923833304	-0.988854937	-0.111930957	FALSE
Apoptosis	Up	0.821	0.832	1	-1.091408326	-1.056358938	0.064861037	FALSE
Intrinsic Pathway for Apoptosis	Up	0.821	0.832	1	-1.091408326	-1.056358938	0.064861037	FALSE
Apoptotic factor-mediated response	Up	0.821	0.832	1	-1.091408326	-1.056358938	0.064861037	FALSE
Intrinsic Pathway of Fibrin Clot Formation	Up	0.821	0.832	1	-1.091408326	-1.056358938	0.064861037	FALSE
Formation of Fibrin Clot (Clotting Cascade)	Up	0.821	0.832	1	-1.091408326	-1.056358938	0.064861037	FALSE
Signaling by Rho GTPases	Up	0.821	0.832	1	-1.091408326	-1.056358938	0.064861037	FALSE
Programmed Cell Death	Up	0.821	0.832	1	-1.091408326	-1.056358938	0.064861037	FALSE
RHOA GTPase cycle	Up	0.821	0.832	1	-1.091408326	-1.056358938	0.064861037	FALSE
RHO GTPase cycle	Up	0.821	0.832	1	-1.091408326	-1.056358938	0.064861037	FALSE
RHOC GTPase cycle	Up	0.821	0.832	1	-1.091408326	-1.056358938	0.064861037	FALSE
Defective Intrinsic Pathway for Apoptosis Due to p14ARF Loss of Function	Up	0.821	0.832	1	-1.091408326	-1.056358938	0.064861037	FALSE
Diseases of programmed cell death	Up	0.821	0.832	1	-1.091408326	-1.056358938	0.064861037	FALSE
Signaling by Rho GTPases, Miro GTPases and RHOBTB3	Up	0.821	0.832	1	-1.091408326	-1.056358938	0.064861037	FALSE
Defective Intrinsic Pathway for Apoptosis	Up	0.821	0.832	1	-1.091408326	-1.056358938	0.064861037	FALSE
Viral Infection Pathways	Down	0.887	0.833	3	-1.07590944	-1.545340619	-0.200929825	FALSE
Signal Transduction	Up	0.892	0.901	2	-1.440749992	-1.407046826	0.073099372	FALSE
Infectious disease	Down	0.945	0.924	4	-1.404824833	-1.805057116	-0.130362942	FALSE

Binding and Uptake of Ligands by Scavenger Receptors	Down	0.939	0.946	2	-1.565797919	-1.575717989	-0.068524104	FALSE
Scavenging by Class A Receptors	Down	0.939	0.946	2	-1.565797919	-1.575717989	-0.068524104	FALSE
Disease	Down	0.979	0.967	5	-1.744606171	-2.086910245	-0.091318146	FALSE

Supplementary Table 5. Serum pathways

Name	Direction	FDR	Pvalue	Number of Genes	MeanAbsT0	MeanWeightT0	av_foldchange	sig.
Binding and Uptake of Ligands by Scavenger Receptors	Up	0.00001	0.00001	3	2.771336262	2.554735003	0.752486974	TRUE
Scavenging by Class A Receptors	Up	0.00001	0.00001	3	2.771336262	2.554735003	0.752486974	TRUE
Vesicle-mediated transport	Up	0.00001	0.00001	5	2.572450277	2.324730191	0.816023449	TRUE
Lectin pathway of complement activation	Up	0.021	0.013	6	1.467752745	1.422546853	0.18673068	FALSE
Creation of C4 and C2 activators	Up	0.056	0.026	7	1.207799432	1.185567077	0.203065165	FALSE
Apoptosis	Down	0.074	0.05	1	0.786926101	0.654764452	-0.502440145	FALSE
Intrinsic Pathway for Apoptosis	Down	0.074	0.05	1	0.786926101	0.654764452	-0.502440145	FALSE
Apoptotic factor-mediated response	Down	0.074	0.05	1	0.786926101	0.654764452	-0.502440145	FALSE
Signaling by Rho GTPases	Down	0.074	0.05	1	0.786926101	0.654764452	-0.502440145	FALSE
RHOA GTPase cycle	Down	0.074	0.05	1	0.786926101	0.654764452	-0.502440145	FALSE
RHO GTPase cycle	Down	0.074	0.05	1	0.786926101	0.654764452	-0.502440145	FALSE
RHOC GTPase cycle	Down	0.074	0.05	1	0.786926101	0.654764452	-0.502440145	FALSE
Defective Intrinsic Pathway for Apoptosis Due to p14ARF Loss of Function	Down	0.074	0.05	1	0.786926101	0.654764452	-0.502440145	FALSE
Diseases of programmed cell death	Down	0.074	0.05	1	0.786926101	0.654764452	-0.502440145	FALSE
Signaling by Rho GTPases, Miro GTPases and RHOBTB3	Down	0.074	0.05	1	0.786926101	0.654764452	-0.502440145	FALSE
Defective Intrinsic Pathway for Apoptosis	Down	0.074	0.05	1	0.786926101	0.654764452	-0.502440145	FALSE
Metabolism	Up	0.162	0.1	1	0.857033773	0.90876556	1.054334072	FALSE
Acyl chain remodelling of PC	Up	0.162	0.1	1	0.857033773	0.90876556	1.054334072	FALSE
Acyl chain remodelling of PS	Up	0.162	0.1	1	0.857033773	0.90876556	1.054334072	FALSE
Acyl chain remodelling of PE	Up	0.162	0.1	1	0.857033773	0.90876556	1.054334072	FALSE
Glycerophospholipid biosynthesis	Up	0.162	0.1	1	0.857033773	0.90876556	1.054334072	FALSE
Phospholipid metabolism	Up	0.162	0.1	1	0.857033773	0.90876556	1.054334072	FALSE
Metabolism of lipids	Up	0.162	0.1	1	0.857033773	0.90876556	1.054334072	FALSE
Class B/2 (Secretin family receptors)	Up	0.158	0.108	1	0.758694732	0.629139917	1.112633748	FALSE
Collagen formation	Up	0.174	0.135	1	0.688978204	0.657889162	0.553687843	FALSE
Trafficking and processing of endosomal TLR	Up	0.174	0.135	1	0.688978204	0.657889162	0.553687843	FALSE
Assembly of collagen fibrils and other multimeric structures	Up	0.174	0.135	1	0.688978204	0.657889162	0.553687843	FALSE
MHC class II antigen presentation	Up	0.174	0.135	1	0.688978204	0.657889162	0.553687843	FALSE
ER to Golgi Anterograde Transport	Up	0.218	0.168	2	0.673221147	0.546822582	0.911328161	FALSE
Membrane Trafficking	Up	0.218	0.168	2	0.673221147	0.546822582	0.911328161	FALSE
COPI-mediated anterograde transport	Up	0.218	0.168	2	0.673221147	0.546822582	0.911328161	FALSE
Transport to the Golgi and subsequent modification	Up	0.218	0.168	2	0.673221147	0.546822582	0.911328161	FALSE
Intrinsic Pathway of Fibrin Clot Formation	Down	0.209	0.187	3	0.268007107	0.045207183	-0.148140114	FALSE
Formation of Fibrin Clot (Clotting Cascade)	Down	0.209	0.187	3	0.268007107	0.045207183	-0.148140114	FALSE
Classical antibody-mediated complement activation	Down	0.261	0.187	2	0.28272672	0.210332007	-0.159094912	FALSE
Asparagine N-linked glycosylation	Up	0.232	0.189	3	0.587750137	0.377823563	0.469199745	FALSE
Programmed Cell Death	Down	0.289	0.216	2	0.253489946	0.167570731	-0.462998371	FALSE
Ficolins bind to repetitive carbohydrate structures on the target cell surface	Down	0.351	0.267	4	0.203667318	0.201131993	-0.446535887	FALSE
COPII-mediated vesicle transport	Up	0.326	0.277	1	0.193383745	0.144183994	0.710022574	FALSE
Cargo concentration in the ER	Up	0.326	0.277	1	0.193383745	0.144183994	0.710022574	FALSE
Terminal pathway of complement	Down	0.356	0.285	6	0.023389735	0.154814517	-0.034320325	FALSE
ER-Phagosome pathway	Down	0.333	0.29	1	0.065934622	-0.118914304	-0.415057087	FALSE
Antigen processing-Cross presentation	Down	0.333	0.29	1	0.065934622	-0.118914304	-0.415057087	FALSE
Influenza Infection	Down	0.333	0.29	1	0.065934622	-0.118914304	-0.415057087	FALSE
Virus Assembly and Release	Down	0.333	0.29	1	0.065934622	-0.118914304	-0.415057087	FALSE
Assembly of Viral Components at the Budding Site	Down	0.333	0.29	1	0.065934622	-0.118914304	-0.415057087	FALSE
Cellular responses to stress	Down	0.333	0.29	1	0.065934622	-0.118914304	-0.415057087	FALSE
Scavenging by Class F Receptors	Down	0.333	0.29	1	0.065934622	-0.118914304	-0.415057087	FALSE
ATF6 (ATF6-alpha) activates chaperones	Down	0.333	0.29	1	0.065934622	-0.118914304	-0.415057087	FALSE
Unfolded Protein Response (UPR)	Down	0.333	0.29	1	0.065934622	-0.118914304	-0.415057087	FALSE
ATF6 (ATF6-alpha) activates chaperone genes	Down	0.333	0.29	1	0.065934622	-0.118914304	-0.415057087	FALSE
N-glycan trimming in the ER and Calnexin/Calreticulin cycle	Down	0.333	0.29	1	0.065934622	-0.118914304	-0.415057087	FALSE
Cellular responses to stimuli	Down	0.333	0.29	1	0.065934622	-0.118914304	-0.415057087	FALSE
Calnexin/calreticulin cycle	Down	0.333	0.29	1	0.065934622	-0.118914304	-0.415057087	FALSE
Class I MHC mediated antigen processing & presentation	Down	0.333	0.29	1	0.065934622	-0.118914304	-0.415057087	FALSE
Antigen Presentation: Folding, assembly and peptide loading of class I MHC	Down	0.333	0.29	1	0.065934622	-0.118914304	-0.415057087	FALSE
Collagen degradation	Up	0.359	0.302	2	0.184230324	0.169780235	0.065065624	FALSE
Defective SERPING1 causes hereditary angioedema	Up	0.356	0.304	1	0.031404426	0.005911768	0.511683492	FALSE
SARS-CoV Infections	Down	0.331	0.329	2	0.037362614	-0.120978815	-0.502257086	FALSE
SARS-CoV-2 Infection	Down	0.331	0.329	2	0.037362614	-0.120978815	-0.502257086	FALSE
SARS-CoV-2 activates/modulates innate and adaptive immune responses	Down	0.331	0.329	2	0.037362614	-0.120978815	-0.502257086	FALSE
SARS-CoV-2-host interactions	Down	0.331	0.329	2	0.037362614	-0.120978815	-0.502257086	FALSE
Hemostasis	Down	0.362	0.383	6	-0.129319634	-0.366571672	-0.133912861	FALSE
Complement cascade	Up	0.487	0.384	28	-0.200182881	-0.128979035	0.041260635	FALSE
Toll-like Receptor Cascades	Up	0.384	0.387	2	-0.040819439	-0.112043455	0.084217784	FALSE
Immune System	Up	0.511	0.388	33	-0.181519349	-0.125477806	0.075772542	FALSE
Innate Immune System	Up	0.514	0.4	32	-0.195989474	-0.106402037	0.091110968	FALSE
Defects of contact activation system (CAS) and kallikrein/kinin system (KKS)	Up	0.452	0.445	2	-0.228200453	-0.407621118	0.029009902	FALSE
Diseases of hemostasis	Up	0.452	0.445	2	-0.228200453	-0.407621118	0.029009902	FALSE
Common Pathway of Fibrin Clot Formation	Down	0.473	0.483	1	-0.354128601	-0.582375082	-0.453663688	FALSE
Gamma-carboxylation of protein precursors	Down	0.473	0.483	1	-0.354128601	-0.582375082	-0.453663688	FALSE
Transport of gamma-carboxylated protein precursors from the endoplasmic reticulum to the Golgi apparatus	Down	0.473	0.483	1	-0.354128601	-0.582375082	-0.453663688	FALSE
Removal of aminoterminal propeptides from gamma-carboxylated proteins	Down	0.473	0.483	1	-0.354128601	-0.582375082	-0.453663688	FALSE
Gamma-carboxylation, transport, and amino-terminal cleavage of proteins	Down	0.473	0.483	1	-0.354128601	-0.582375082	-0.453663688	FALSE
Gamma carboxylation, hypusine formation and arylsulfatase activation	Down	0.473	0.483	1	-0.354128601	-0.582375082	-0.453663688	FALSE
Cell surface interactions at the vascular wall	Down	0.473	0.483	1	-0.354128601	-0.582375082	-0.453663688	FALSE
G alpha (q) signalling events	Down	0.473	0.483	1	-0.354128601	-0.582375082	-0.453663688	FALSE
Thrombin signalling through proteinase activated receptors (PARs)	Down	0.473	0.483	1	-0.354128601	-0.582375082	-0.453663688	FALSE
Defective factor XII causes hereditary angioedema	Down	0.473	0.483	1	-0.354128601	-0.582375082	-0.453663688	FALSE
Defective factor VIII causes hemophilia A	Down	0.473	0.483	1	-0.354128601	-0.582375082	-0.453663688	FALSE
Defective F8 cleavage by thrombin	Down	0.473	0.483	1	-0.354128601	-0.582375082	-0.453663688	FALSE
Antimicrobial peptides	Down	0.544	0.51	3	-0.32668152	-0.327368695	-0.248330651	FALSE
Platelet degranulation	Up	0.489	0.511	4	-0.374782309	-0.48515146	0.038156667	FALSE
Response to elevated platelet cytosolic Ca2+	Up	0.489	0.511	4	-0.374782309	-0.48515146	0.038156667	FALSE
Degradation of the extracellular matrix	Up	0.543	0.522	3	-0.301568236	-0.322584269	0.149385816	FALSE
Regulated Necrosis	Down	0.559	0.522	1	-0.428437182	-0.417783651	-0.423556596	FALSE
Pyroptosis	Down	0.559	0.522	1	-0.428437182	-0.417783651	-0.423556596	FALSE
Elastic fibre formation	Down	0.58	0.553	1	-0.487656187	-0.460650841	-0.370550339	FALSE
Molecules associated with elastic fibres	Down	0.58	0.553	1	-0.487656187	-0.460650841	-0.370550339	FALSE
Syndecan interactions	Down	0.58	0.553	1	-0.487656187	-0.460650841	-0.370550339	FALSE
Non-integrin membrane-ECM interactions	Down	0.58	0.553	1	-0.487656187	-0.460650841	-0.370550339	FALSE
Platelet activation, signaling and aggregation	Down	0.556	0.615	5	-0.493586613	-0.694378712	-0.060207404	FALSE
Developmental Biology	Up	0.564	0.632	1	-0.601210605	-0.842714895	0.413541782	FALSE
Generic Transcription Pathway	Up	0.564	0.632	1	-0.601210605	-0.842714895	0.413541782	FALSE
Integrin signaling	Up	0.564	0.632	1	-0.601210605	-0.842714895	0.413541782	FALSE
GRB2:SOS provides linkage to MAPK signaling for Integrins	Up	0.564	0.632	1	-0.601210605	-0.842714895	0.413541782	FALSE
p130Cas linkage to MAPK signaling for integrins	Up	0.564	0.632	1	-0.601210605	-0.842714895	0.413541782	FALSE
L1CAM interactions	Up	0.564	0.632	1	-0.601210605	-0.842714895	0.413541782	FALSE
Axon guidance	Up	0.564	0.632	1	-0.601210605	-0.842714895	0.413541782	FALSE
Signal transduction by L1	Up	0.564	0.632	1	-0.601210605	-0.842714895	0.413541782	FALSE
Diseases of signal transduction by growth factor receptors and second messengers	Up	0.564	0.632	1	-0.601210605	-0.842714895	0.413541782	FALSE
RAF/MAP kinase cascade	Up	0.564	0.632	1	-0.601210605	-0.842714895	0.413541782	FALSE
MAP2K and MAPK activation	Up	0.564	0.632	1	-0.601210605	-0.842714895	0.413541782	FALSE
MAPK family signaling cascades	Up	0.564	0.632	1	-0.601210605	-0.842714895	0.413541782	FALSE
MAPK1/MAPK3 signaling	Up	0.564	0.632	1	-0.601210605	-0.842714895	0.413541782	FALSE
Signaling by moderate kinase activity BRAF mutants	Up	0.564	0.632	1	-0.601210605	-0.842714895	0.413541782	FALSE
Signaling by high-kinase activity BRAF mutants	Up	0.564	0.632	1	-0.601210605	-0.842714895	0.413541782	FALSE
Signaling by RAS mutants	Up	0.564	0.632	1	-0.601210605	-0.842714895	0.413541782	FALSE
Signaling by BRAF and RAF1 fusions	Up	0.564	0.632	1	-0.601210605	-0.842714895	0.413541782	FALSE
Paradoxical activation of RAF signaling by kinase inactive BRAF	Up	0.564	0.632	1	-0.601210605	-0.842714895	0.413541782	FALSE
Oncogenic MAPK signaling	Up	0.564	0.632	1	-0.601210605	-0.842714895	0.413541782	FALSE
RNA Polymerase II Transcription	Up	0.564	0.632	1	-0.601210605	-0.842714895	0.413541782	FALSE
Gene expression (Transcription)	Up	0.564	0.632	1	-0.601210605	-0.842714895	0.413541782	FALSE
Transcriptional regulation by RUNX1	Up	0.564	0.632	1	-0.601210605	-0.842714895	0.413541782	FALSE
RUNX1 regulates genes involved in megakaryocyte differentiation and platelet function	Up	0.564	0.632	1	-0.601210605	-0.842714895	0.413541782	FALSE
Signaling downstream of RAS mutants	Up	0.564	0.632	1	-0.601210605	-0.842714895	0.413541782	FALSE
Signaling by RAF1 mutants	Up	0.564	0.632	1	-0.601210605	-0.842714895	0.413541782	FALSE
Nervous system development	Up	0.564	0.632	1	-0.601210605	-0.842714895	0.413541782	FALSE
Signal Transduction	Up	0.551	0.637	6	-0.495216174	-0.774557553	0.083003165	FALSE
Initial triggering of complement	Up	0.682	0.658	13	-0.598118582	-0.549999464	0.098300275	FALSE
Neutrophil degranulation	Up	0.666	0.661	10	-0.534146761	-0.524948224	0.314708707	FALSE
Diseases of glycosylation	Up	0.671	0.67	1	-0.803552468	-0.788852151	0.192543952	FALSE
Diseases associated with O-glycosylation of proteins	Up	0.671	0.67	1	-0.803552468	-0.788852151	0.192543952	FALSE
Defective B3GALTL causes PpS	Up	0.671	0.67	1	-0.803552468	-0.788852151	0.192543952	FALSE
O-linked glycosylation	Up	0.671	0.67	1	-0.803552468	-0.788852151	0.192543952	FALSE
O-glycosylation of TSR domain-containing proteins	Up	0.671	0.67	1	-0.803552468	-0.788852151	0.192543952	FALSE
Regulation of Complement cascade	Down	0.734	0.67	22	-0.645940617	-0.583882588	-0.020509598	FALSE

Cell-Cell communication	Down	0.66	0.679	1	-0.746705608	-0.816342535	-0.385252276	FALSE
Toll Like Receptor 4 (TLR4) Cascade	Down	0.66	0.679	1	-0.746705608	-0.816342535	-0.385252276	FALSE
Toll Like Receptor TLR1:TLR2 Cascade	Down	0.66	0.679	1	-0.746705608	-0.816342535	-0.385252276	FALSE
Toll Like Receptor 2 (TLR2) Cascade	Down	0.66	0.679	1	-0.746705608	-0.816342535	-0.385252276	FALSE
Signal regulatory protein family interactions	Down	0.66	0.679	1	-0.746705608	-0.816342535	-0.385252276	FALSE
Surfactant metabolism	Down	0.66	0.679	1	-0.746705608	-0.816342535	-0.385252276	FALSE
Regulation of TLR by endogenous ligand	Down	0.66	0.679	1	-0.746705608	-0.816342535	-0.385252276	FALSE
Diseases associated with surfactant metabolism	Down	0.66	0.679	1	-0.746705608	-0.816342535	-0.385252276	FALSE
Defective CSF2RB causes SMDP5	Down	0.66	0.679	1	-0.746705608	-0.816342535	-0.385252276	FALSE
Defective CSF2RA causes SMDP4	Down	0.66	0.679	1	-0.746705608	-0.816342535	-0.385252276	FALSE
SARS-CoV-1 Infection	Down	0.66	0.679	1	-0.746705608	-0.816342535	-0.385252276	FALSE
SARS-CoV-1-host interactions	Down	0.66	0.679	1	-0.746705608	-0.816342535	-0.385252276	FALSE
SARS-CoV-1 activates/modulates innate immune responses	Down	0.66	0.679	1	-0.746705608	-0.816342535	-0.385252276	FALSE
Signaling by GPCR	Up	0.71	0.719	4	-0.699371218	-0.854660169	0.146729338	FALSE
GPCR ligand binding	Up	0.71	0.719	4	-0.699371218	-0.854660169	0.146729338	FALSE
Cytokine Signaling in Immune system	Up	0.713	0.723	1	-0.782872529	-0.798837855	0.318026202	FALSE
Metabolism of Angiotensinogen to Angiotensins	Up	0.713	0.723	1	-0.782872529	-0.798837855	0.318026202	FALSE
Peptide hormone metabolism	Up	0.713	0.723	1	-0.782872529	-0.798837855	0.318026202	FALSE
Interleukin-1 family signaling	Up	0.713	0.723	1	-0.782872529	-0.798837855	0.318026202	FALSE
Interleukin-1 processing	Up	0.713	0.723	1	-0.782872529	-0.798837855	0.318026202	FALSE
Signaling by Interleukins	Up	0.713	0.723	1	-0.782872529	-0.798837855	0.318026202	FALSE
Suppression of apoptosis	Up	0.713	0.723	1	-0.782872529	-0.798837855	0.318026202	FALSE
Infection with Mycobacterium tuberculosis	Up	0.713	0.723	1	-0.782872529	-0.798837855	0.318026202	FALSE
Response of Mtb to phagocytosis	Up	0.713	0.723	1	-0.782872529	-0.798837855	0.318026202	FALSE
Platelet Aggregation (Plug Formation)	Down	0.668	0.737	2	-0.675526831	-1.007690786	-0.020060953	FALSE
C-type lectin receptors (CLRs)	Down	0.745	0.742	1	-0.955232736	-0.901155274	-0.127167833	FALSE
CD209 (DC-SIGN) signaling	Down	0.745	0.742	1	-0.955232736	-0.901155274	-0.127167833	FALSE
Butyrophilin (BTN) family interactions	Down	0.745	0.742	1	-0.955232736	-0.901155274	-0.127167833	FALSE
Infectious disease	Down	0.71	0.744	5	-0.76299387	-0.944492514	-0.207245549	FALSE
Integrin cell surface interactions	Up	0.704	0.75	2	-0.769945093	-0.92161875	0.021495722	FALSE
ECM proteoglycans	Up	0.704	0.75	2	-0.769945093	-0.92161875	0.021495722	FALSE
Extracellular matrix organization	Up	0.722	0.759	5	-0.720549784	-0.832755578	0.098229778	FALSE
Activation of Matrix Metalloproteinases	Down	0.776	0.781	2	-0.85652531	-0.860281317	-0.052765197	FALSE
Adaptive Immune System	Down	0.78	0.797	5	-0.88952237	-0.984599037	-0.061694408	FALSE
Post-translational protein modification	Up	0.818	0.818	7	-0.880716398	-1.072630806	0.175138276	FALSE
Disease	Down	0.763	0.834	10	-0.83694969	-1.15904287	-0.087456235	FALSE
Diseases of metabolism	Down	0.83	0.857	2	-1.096197999	-1.135044048	-0.096354162	FALSE
G alpha (i) signalling events	Down	0.864	0.891	2	-1.275131716	-1.241739735	-0.036026354	FALSE
Class A/1 (Rhodopsin-like receptors)	Down	0.872	0.893	3	-1.24559693	-1.350110658	-0.175238798	FALSE
Peptide ligand-binding receptors	Down	0.872	0.893	3	-1.24559693	-1.350110658	-0.175238798	FALSE
GPCR downstream signalling	Down	0.872	0.893	3	-1.24559693	-1.350110658	-0.175238798	FALSE
Immunoregulatory interactions between a Lymphoid and a non-Lymphoid cell	Down	0.88	0.91	2	-1.264810793	-1.300687541	-0.159967483	FALSE
Leishmania infection	Up	0.894	0.914	2	-1.290384667	-1.288309863	0.191671756	FALSE
Purinergic signaling in leishmaniasis infection	Up	0.894	0.914	2	-1.290384667	-1.288309863	0.191671756	FALSE
Cell recruitment (pro-inflammatory response)	Up	0.894	0.914	2	-1.290384667	-1.288309863	0.191671756	FALSE
Metabolism of proteins	Up	0.918	0.934	9	-1.286578234	-1.484364917	0.128749096	FALSE
Post-translational protein phosphorylation	Up	0.937	0.95	2	-1.548909339	-1.499841885	0.039744217	FALSE
Alternative complement activation	Down	0.94	0.956	4	-1.628731517	-1.564894747	-0.022354362	FALSE
Regulation of Insulin-like Growth Factor (IGF) transport and uptake by Insulin-like Growth Factor Binding Protein	Down	0.97	0.979	4	-1.663744862	-1.751154835	-0.014037263	FALSE
Activation of C3 and C5	Down	0.98	0.989	6	-2.158876805	-2.069672916	-0.024630925	FALSE

Supplementary Table 6. Spearman's correlation of serum complement proteins with MELD & albumin in patients with sAH: significant findings only

**MELD**		
**Complement proteins**	**Correlation coefficient (r)**	***p*. value**
C1QBP	-0.77	0.0002
CD59	0.77	0.0002
ITGα2/β3	-0.66	0.003
MASP1	-0.65	0.003
CD93	0.59	0.01
C8αβγ	-0.58	0.01
CD55	0.58	0.01
FCN1	-0.57	0.01
CFD	0.55	0.02
F2	-0.49	0.04
CTSG	0.48	0.05
FCN3	0.47	0.05
**Albumin**		
**Complement proteins**	**Correlation coefficient (r)**	***p*. value**
FCN1	-0.57	0.01
C1QBP	-0.56	0.02
ITGα2/β3	-0.55	0.02
CD93	0.47	0.05

MELD, Model for End-Stage Liver Disease

Supplementary Table 7. Albumin infusion status in patients with severe AH

Sample ID	Albumin infusion	Albumin infusion day (0=sample collection)	Index Albumin
672	No		2.3
559	No		2.1
569	No		2.6
557	Yes	-6, 0	3.5
635	No		2.7
607	Yes	-3,-2, 0	3.7
601	Yes	-4,-3, 0	4
515	No		2.8
605	Yes	-7, -5	2.6
473	Yes	-14, -8,-3, 0	2.6
673	No		3.3
636	Yes	-13-12-11-1	2.5
482	Yes	-11, 0	2.6
676	No		2.8
442	Yes	-4	2.8
677	No		2.2
432	Yes	-2	3.2
431	Yes	-13	3

Supplementary Table 8. Point-biserial correlation analysis between serum complement proteins and severe AH: significant findings only

Complement Proteins	Coefficient (r)	95% CI	*p*. value
C1QBP	-0.87	-0.941 to -0.714	<0.0001
C7	0.81	0.596 to 0.913	<0.0001
CD55	0.71	0.434 to 0.867	<0.0001
CD93	0.81	0.601 to 0.914	<0.0001
CFHR5	-0.83	-0.922 to -0.634	<0.0001
CFI	-0.78	-0.899 to -0.544	<0.0001
CLU	-0.81	-0.914 to -0.603	<0.0001
COLEC11	0.82	0.624 to 0.920	<0.0001
CTSB	0.75	0.497 to 0.886	<0.0001
FCN2	-0.72	-0.871 to -0.446	<0.0001
MASP1	-0.79	-0.903 to -0.560	<0.0001
SERPING1	0.73	0.471 to 0.878	<0.0001
C8αβγ	-0.65	-0.837 to -0.340	0.0005
C1R	0.63	0.305 to 0.824	0.001
CFH	0.59	0.248 to 0.804	0.0023
CD59	0.59	0.243 to 0.802	0.0025
C1QABC	0.52	0.150 to 0.764	0.0089
CALR	-0.50	-0.749 to -0.115	0.0138
CFP	0.42	0.023 to 0.706	0.0396

CI, Confidence Interval

Supplementary Table 9. Clinical features of patients with sAH (serum proteome - Test cohort 2)

	Severe AH (sAH)		
**Clinical features**	**Alive (n = 7)**	**Dead (n = 11)**	***p.* value**
Age, mean (SE)	43.6 (2.9)	47.8 (2.4)	0.211
Male, n (%)	5 (71)	8 (72)	>1.000
BMI, mean (SE)	33.8 (3.1)	29.7 (2.0)	0.260
Albumin (g/dL), mean (SE)	2.6 (0.1)	3.0 (0.2)	0.082
MELD	25 (2.1)	36 (2.6)	0.004
Creatinine (mg/dL), mean (SE)	1.72 (0.7)	2.84 (0.5)	0.126
eGFR (ml/min/1.73m^2), mean (SE)	83.14 (18.8)	41.45 (10.4)	0.058

eGFR, Estimated Glomerular Filtration Rate. eGFR was calculated using the 2021 CKD-EPI equations for GFR from MDCalc (https://www.mdcalc.com/calc/3939/ckd-epi-equations-glomerular-filtration-rate-gfr)

Supplementary Table 10. LOOCV ROC curve analysis of complement proteins significantly changed in liver proteome

Protein	AUC	Optimal threshold	Sensitivity %	Specificity %
CD59	1.00	0.50	100.0	100.0
C1QR1	1.00	0.44	100.0	100.0
FCN1	0.94	0.00	100.0	83.3
MBL2	0.92	0.38	100.0	91.7
C4BPB	0.92	0.21	100.0	91.7
FCN2	0.89	0.09	100.0	66.7
C1QA	0.88	0.24	83.3	83.3
C1QC	0.86	0.28	83.3	91.7
CD209	0.86	0.32	100.0	83.3
C1QB	0.81	0.23	100.0	66.7
C4BPA	0.79	0.34	83.3	83.3
FCN3	0.78	0.62	66.7	100.0
CLU	0.76	0.52	83.3	91.7

LOOCV, Leave-One-Out Cross-Validation; ROC, Receiver Operating Characteristic; AUC, Area Under Curve

Supplementary Table 11. LOOCV ROC curve analysis of serum complement proteins with significant changes: only proteins with AUC ≥ 0.70 included

sAH vs HC

Protein	AUC	Optimal threshold	Sensitivity (%)	Specificity (%)
C1QBP	1.00	0.50	100.0	100.0
COLEC11	1.00	0.50	100.0	100.0
CD59	1.00	0.68	100.0	100.0
CD55	1.00	0.50	100.0	100.0
CFHR5	1.00	0.61	100.0	100.0
C7	0.99	0.85	94.4	100.0
SERPING1	0.99	1.00	94.4	100.0
CFI	0.94	0.74	94.4	100.0
CTSB	0.94	0.72	94.4	100.0
CLU	0.94	0.86	88.9	100.0
FCN2	0.92	0.91	83.3	100.0
CALR	0.92	0.74	88.9	100.0
CFH	0.91	0.83	83.3	100.0
C8αβγ	0.89	0.93	66.7	100.0
C1QABC	0.85	0.68	83.3	83.3
CD93	0.82	0.57	94.4	83.3
MASP1	0.79	0.42	94.4	83.3
C1R	0.79	0.57	94.4	83.3
CFP	0.78	0.69	72.2	83.3

sAH vs AUD

Protein	AUC	Optimal threshold	Sensitivity (%)	Specificity (%)
C1QBP	1.00	0.50	100.0	100.0
COLEC11	1.00	0.49	100.0	100.0
CD59	0.97	0.81	88.9	100.0
MASP1	0.95	0.25	94.4	85.0
C8αβγ	0.95	0.21	100.0	90.0
CD55	0.94	0.14	100.0	85.0
FCN2	0.94	0.55	88.9	95.0
C7	0.94	0.63	88.9	100.0
CFI	0.94	0.55	94.4	95.0
CD93	0.93	0.58	88.9	95.0
CLU	0.93	0.51	88.9	90.0
CFHR5	0.92	0.41	100.0	80.0
CALR	0.92	0.60	88.9	95.0
C1QABC	0.91	0.40	88.9	80.0
SERPING1	0.86	0.41	83.3	80.0

C9	0.86	0.44	88.9	80.0
CTSB	0.84	0.37	94.4	85.0
CFH	0.84	0.71	72.2	100.0
CFB	0.82	0.35	83.3	70.0
C6	0.79	0.60	66.7	85.0
CFP	0.79	0.52	66.7	95.0
ELANE	0.71	0.56	66.7	80.0

LOOCV, Leave-One-Out Cross-Validation; ROC, Receiver Operating Characteristic; AUC, Area Under Curve

Supplementary Table 12. Serum complement proteins in compensated vs decompensated AC

Protein	Compensated	Decompensated	*p*. value	adj. *p. value*
C1QBP	0.927	0.775	0.0031	0.11
COLEC11	8.674	9.382	0.8133	0.84
C7	2.273	3.942	0.014	0.25
CD59	1.240	1.049	0.1918	0.41
CD55	1.653	1.748	0.7214	0.79
FCN2	0.853	0.726	0.2302	0.41
CD93	1.781	1.864	0.7867	0.83
MASP1	0.924	0.742	0.1485	0.41
CFI	0.880	0.760	0.0554	0.33
C8αβγ	1.099	1.024	0.6557	0.76
CLU	0.711	0.763	0.5683	0.73
CFB	1.111	0.989	0.1537	0.41
CTSB	1.476	1.346	0.423	0.61
C1QABC	1.054	1.153	0.2996	0.48
CFHR5	0.610	0.459	0.1941	0.41
CALR	1.085	0.806	0.0734	0.38
CFH	1.014	1.108	0.1135	0.41
CFP	1.073	1.085	0.8556	0.86
SERPING1	1.217	1.329	0.5432	0.72
C9	1.336	1.135	0.0444	0.33
C1R	2.074	2.661	0.3077	0.48
C6	0.948	1.104	0.203	0.41
FCN3	0.775	0.612	0.2789	0.48
CTSG	1.036	1.403	0.1171	0.41
C5	0.897	1.012	0.1628	0.41
MBL2	1.088	1.312	0.541	0.72
CD209	0.850	0.751	0.3251	0.49
CFD	0.992	0.860	0.2291	0.41
ELANE	0.828	0.753	0.6833	0.77
VTN	1.087	0.687	0.0292	0.33
C2	0.981	0.932	0.1672	0.41
ITGα2/β3	1.485	2.078	0.0836	0.38
FCN1	0.889	0.936	0.6562	0.76
C4AB	0.614	1.127	0.2022	0.41
C3	1.254	1.177	0.6444	0.76
F2	1.037	0.532	0.0487	0.33

No significantly changed proteins between compensated (AC.c, mean of n=7) and decompensated AC (AC.d, mean of n=6). adj. *p*. value, Benjamini-Hochberg adj. *p*. value

Supplementary Table 13. Clinical features of patients used in the validation cohorts

A. Immunoblotting (Validation Cohort 1)

	n	Age, years (mean/SE)	Sex	MELD (mean/SE)
Healthy controls	5	N/A	N/A	N/A
Severe AH	5	42 (4.2)	3M/2F	44(9.0)

B. COLEC11 ELISA (Validation Cohort 2)

Variables	Healthy Control (HC)	Severe AH (sAH)
No. of cases	23	110
**Age (years) mean (SE)**	41 (3.3)	47 (0.9)
**Male, n (%)**	10 (43.5)	73 (66.4)
**BMI (kg/m^2), mean (SE)**	ND	30 (1.1)
**Race, n (%)**		
White	8 (34.8)	98 (89.1)
African/American	0	10 (9.1)
American Indian	0	1 (0.9)
Asian	0	0
Other	0	1 (0.9)
Unknown	15 (65.2)	0
**Liver function score, mean (SE)**		
MELD	ND	26 (0.5)
Maddrey score	ND	59 (2.4)
**Laboratory Tests, mean (SE)**		
AST (IU/L)	ND	123 (6.7)
ALT (IU/L)	ND	49 (3.4)
Total Bilirubin (mg/dL)	ND	18 (0.8)
Albumin (g/dL)	ND	3 (0.1)
Creatinine (mg/dL)	ND	1 (0.1)
INR	ND	2 (0.04)

C. MASP1 and F2 ELISA (Validation Cohort 3)

Variables	Healthy Control (HC)	Severe AH (sAH)	Alcohol Cirrhosis (AC)
N0. of cases	25	98	17
**Age (years) mean (SE)**	45 (2.2)	45 (1.0)	52 (2.4)
**Male, n (%)**	12 (48.0)	66 (67.3)	11 (64.7)
**BMI (kg/m^2), mean (SE)**	26.9 (1.0)	30.5 (0.7)	28.5 (2.1)
**Race, n (%)**			
White	17 (68.0)	86 (87.8)	16 (94.1)

African/American	3 (12.0)	8 (8.2)	1 (5.9)
American Indian	0	1 (1.0)	0
Asian	5 (20.0)	0	0
Other	0	0	0
Unknown	0	3 (3.0)	0
**Liver function score, mean (SE)**			
MELD	7 (0.2)	30 (0.8)	18 (2.8)
Child-pugh score	5 (0.0)	10 (0.2)	8 (1.5)
**Laboratory Tests, mean (SE)**			
AST (IU/L)	22 (2.0)	131 (8.4)	52 (4.2)
ALT (IU/L)	21 (2.4)	57 (4.2)	26 (3.0)
Total Bilirubin (mg/dL)	0.5 (0.05)	22.0 (1.2)	7.0 (2.4)
Albumin (g/dL)	4.4 (0.04)	3.0 (0.1)	3.3 (0.3)
Creatinine (mg/dL)	0.86 (0.03)	1.96 (0.2)	1.28 (0.2)
INR	1.02 (0.02)	2.0 (0.1)	1.7 (0.3)

ND, Not Done; MELD, Model for End-Stage Liver Disease; BMI, Body Mass Index; AST, Aspartate aminotransferase; ALT, Alanine transaminase; INR, International Normalized Ratio

Supplemental Table 14. Spearman's correlation analysis of complement proteins and pro-inflammatory cytokines in sAH: Test cohort 3

	Pro-inflammatory cytokines																
**Complement Proteins**	**IL33**	**CXCL1**	**IL17E**	**TSLP**	**S100B**	**IFNα**	**IL12**	**IL18**	**TNFα**	**IL8**	**IL17C**	**IL1β**	**IL1α**	**IFNγ**	**IL17**	**CCL20**	**CX3CL1**
**C7**	-0.37	**-0.78**	-0.49	**-0.68**	-0.15	**-0.86**	**-0.67**	**-0.72**	-0.18	-0.40	-0.27	-0.41	-0.33	-0.37	-0.42	-0.26	-0.40
**VSIG4**	-0.22	-0.13	-0.19	-0.09	-0.07	-0.12	-0.03	0.13	0.20	0.23	0.25	0.29	0.25	0.15	0.47	0.40	0.21
**F2**	0.02	0.02	0.19	-0.22	0.41	0.10	0.04	-0.33	-0.33	-0.49	-0.63	-0.39	**-0.66**	-0.24	-0.59	-0.44	-0.47
**COLEC11**	0.27	-0.23	-0.52	-0.21	-0.30	0.09	-0.57	-0.03	0.39	0.18	0.01	0.14	0.25	0.06	0.23	0.27	**0.70**
**CD59**	0.29	0.26	-0.02	0.14	-0.13	**0.64**	-0.10	0.33	0.21	0.18	-0.22	0.14	0.15	-0.13	0.07	0.23	**0.68**
**VTN**	0.00	-0.22	-0.09	-0.26	0.11	0.03	-0.26	-0.08	-0.34	-0.22	-0.29	-0.13	-0.48	-0.34	-0.17	-0.21	-0.40
**C6**	-0.48	-0.58	-0.20	-0.50	0.03	-0.54	-0.17	-0.43	-0.45	-0.33	-0.12	-0.24	-0.43	-0.26	-0.02	-0.21	-0.62
**CFI**	-0.31	-0.26	-0.41	-0.30	-0.03	**-0.68**	-0.53	-0.09	0.48	0.27	0.09	0.26	0.20	0.21	-0.15	0.36	-0.15
**C4BPA**	0.46	**0.70**	0.52	0.56	0.43	0.51	0.33	0.38	0.31	0.28	-0.07	0.24	0.19	0.24	-0.14	0.18	0.34
**FCN2**	**0.79**	0.58	0.59	0.61	0.36	**0.71**	0.26	0.27	-0.01	-0.01	-0.14	-0.03	0.01	-0.18	-0.16	-0.12	0.47
**SERPING1**	0.02	0.22	0.41	0.26	0.63	-0.17	0.12	0.12	0.24	0.36	0.04	0.34	0.20	0.15	0.11	0.47	0.13
**C8A**	0.10	0.11	-0.38	0.01	-0.19	-0.01	-0.46	0.39	**0.66**	0.44	0.16	0.46	0.37	0.21	0.03	0.47	0.31
**COLEC10**	0.21	0.49	0.18	0.34	-0.01	0.37	-0.05	0.36	0.47	0.40	-0.18	0.22	0.39	0.04	-0.24	0.35	0.62
**CFHR5**	-0.09	0.37	0.09	0.17	0.19	0.25	0.42	0.39	0.25	0.37	0.28	0.46	0.17	**0.67**	0.45	0.33	-0.08
**C8G**	0.07	-0.13	-0.28	-0.18	0.19	-0.18	-0.41	0.14	0.23	0.29	0.10	0.37	-0.06	0.25	0.14	0.28	-0.22
**CD93**	-0.18	0.02	0.10	0.30	-0.11	-0.26	0.30	0.25	0.14	0.43	**0.70**	0.36	0.57	0.31	**0.65**	0.40	0.16
**C8B**	0.45	0.37	0.00	0.19	0.22	0.41	-0.15	0.41	0.35	0.30	-0.02	0.36	0.02	0.28	-0.02	0.20	0.12
**FCN3**	0.44	0.22	0.22	0.35	0.24	0.21	-0.12	0.25	0.33	0.20	0.05	0.23	0.29	-0.19	0.08	0.33	0.62
**CFHR4**	-0.41	-0.32	**-0.72**	-0.49	-0.63	-0.09	-0.18	-0.04	0.18	-0.09	0.13	0.02	0.05	0.25	0.24	0.01	-0.04
**CPN1**	0.33	0.59	0.47	0.52	0.58	0.32	0.25	0.60	0.28	0.44	0.17	0.53	0.12	0.28	0.11	0.37	-0.07
**C1QA**	-0.49	-0.57	**-0.69**	-0.49	-0.60	**-0.71**	-0.52	-0.30	0.20	0.18	0.22	0.01	0.27	0.20	0.07	0.15	-0.05
**CLU**	0.19	0.09	0.07	0.11	-0.26	0.16	0.00	-0.15	-0.04	-0.26	-0.20	-0.37	0.04	-0.29	-0.39	-0.32	0.29
**CFB**	0.18	0.63	0.46	0.57	0.20	0.25	0.20	0.44	0.46	0.41	-0.01	0.28	0.45	0.04	-0.24	0.37	0.45
**C1QB**	-0.57	-0.56	-0.58	-0.44	-0.44	**-0.83**	-0.46	-0.24	0.17	0.24	0.29	0.10	0.25	0.19	0.12	0.22	-0.22
**CFH**	-0.24	0.54	0.30	0.38	0.19	0.05	0.13	0.58	0.61	0.46	-0.01	0.46	0.44	0.01	-0.19	0.62	0.24
**CPN2**	-0.26	0.39	0.30	0.14	0.35	-0.05	0.12	0.22	0.30	0.04	-0.30	0.12	-0.02	-0.13	-0.52	0.19	-0.20
**C1QC**	-0.43	-0.55	**-0.64**	-0.42	-0.53	**-0.71**	-0.46	-0.24	0.15	0.22	0.35	0.08	0.27	0.26	0.20	0.15	-0.13
**CFHR1**	0.62	0.16	0.10	0.26	0.32	0.41	0.06	0.24	0.13	0.22	0.28	0.33	0.11	0.29	0.58	0.22	0.42

**CFP**	-0.43	**-0.67**	-0.34	-0.45	-0.12	**-0.81**	-0.23	-0.48	-0.26	-0.13	0.26	-0.13	-0.11	0.04	0.20	-0.10	-0.51
**CR1L**	-0.16	-0.45	-0.03	-0.54	0.10	-0.09	-0.11	**-0.72**	**-0.72**	**-0.72**	**-0.70**	**-0.71**	**-0.80**	-0.48	-0.48	**-0.67**	-0.47
**C4BPB**	0.13	0.55	0.50	0.27	0.42	0.56	0.46	0.21	-0.06	-0.16	-0.40	-0.06	-0.26	-0.08	-0.32	-0.12	-0.06
**MASP1**	-0.03	0.01	-0.19	0.06	-0.57	0.24	-0.12	0.14	0.19	0.05	-0.01	-0.06	0.38	-0.29	0.05	0.13	**0.70**
**C4A**	0.43	0.26	0.18	0.09	0.22	**0.66**	0.31	0.02	-0.23	-0.27	-0.20	-0.13	-0.35	0.12	0.07	-0.32	0.03
**C9**	-0.18	-0.19	-0.21	-0.24	0.05	-0.06	0.09	-0.02	-0.13	0.10	0.20	0.18	-0.13	0.41	0.47	0.08	-0.31
**C4B**	0.13	0.49	0.45	0.25	0.44	0.33	0.22	0.13	0.15	0.00	-0.43	0.00	-0.09	-0.07	-0.43	0.06	0.13
**C1R**	-0.27	-0.40	**-0.68**	-0.43	**-0.75**	-0.06	-0.44	-0.15	0.18	0.07	-0.04	-0.07	0.26	-0.02	0.16	0.13	0.51
**C5**	0.22	0.00	0.16	-0.02	0.60	-0.32	-0.34	-0.15	0.15	-0.02	-0.25	0.05	-0.22	-0.12	-0.43	0.05	-0.22
**CFD**	-0.04	-0.18	-0.14	-0.10	0.02	-0.05	0.09	-0.04	-0.01	0.10	0.28	0.17	0.10	0.29	0.56	0.18	0.11
**CTSG**	-0.21	0.15	0.16	0.28	0.15	-0.16	0.23	0.42	0.20	**0.64**	0.53	0.57	0.44	0.42	0.58	0.58	-0.01
**MBL2**	-0.53	-0.19	0.03	-0.09	-0.04	**-0.74**	-0.09	-0.25	0.12	0.07	0.03	-0.07	0.23	-0.12	-0.29	0.15	-0.16
**C1S**	-0.29	-0.17	-0.55	-0.14	-0.63	-0.34	-0.42	0.12	0.57	0.47	0.29	0.28	**0.64**	0.25	0.15	0.46	0.47
**C2**	0.38	**0.75**	0.40	**0.68**	0.08	**0.66**	0.42	**0.70**	0.37	0.61	0.27	0.48	0.51	0.42	0.28	0.40	0.53
**CALR**	0.35	0.49	0.14	0.44	0.34	0.38	0.09	**0.70**	0.63	0.63	0.38	**0.76**	0.46	0.45	0.54	**0.72**	0.49
**CFHR2**	0.38	0.32	-0.06	0.32	0.10	0.36	-0.10	0.58	0.61	**0.65**	0.36	**0.67**	0.52	0.42	0.58	**0.69**	**0.68**
**C3**	-0.12	0.43	0.27	0.15	0.23	0.17	0.04	0.24	0.26	0.05	-0.44	0.06	-0.04	-0.19	-0.59	0.14	-0.01

R coefficients are given. Correlations with $p < 0.05$ are shown in color (blue for positive correlations and red for negative correlations).

Supplementary Figure 1

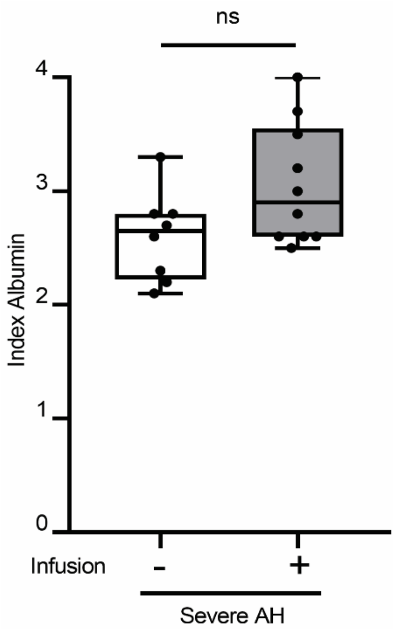

Supplementary Figure 1. Index albumin values in patients with sAH (Test cohort 1): A comparison between those who did not receive albumin infusion (n=8) and those who did (n=10), at least once within the 14 days leading up to the blood draw. Box-and-whisker plots show minimum and maximum values, lower and upper quartiles, and median, with each individual value represented as a point superimposed on the graph. ns - non significant.

Supplementary Figure 2

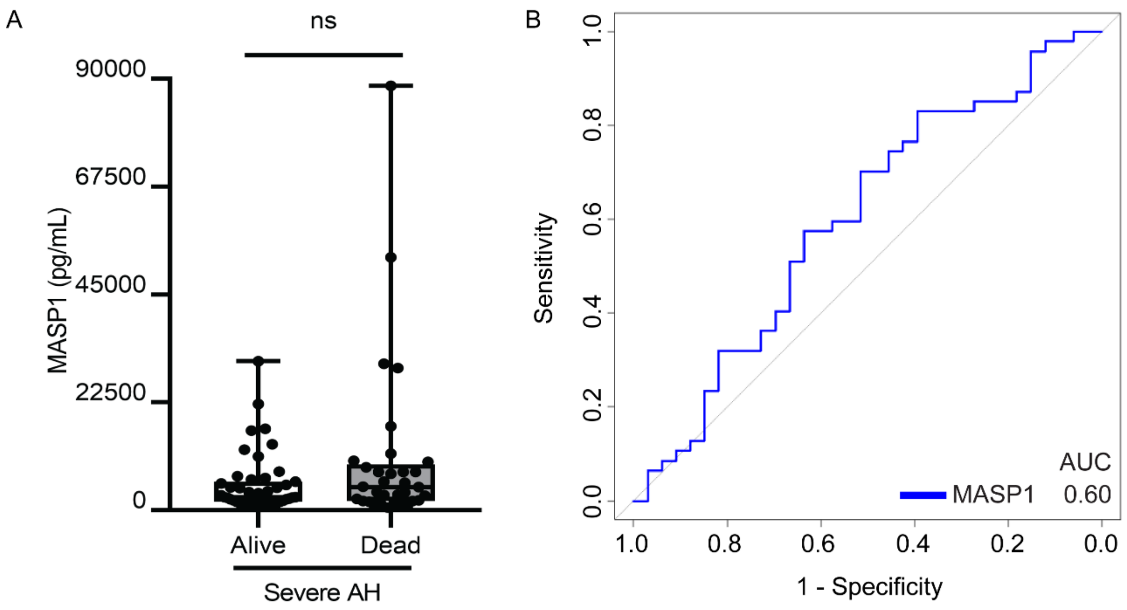

Supplementary Figure 2. MASP1 in patients with sAH based on 90-day survival status: alive vs dead (validation cohort 3). **(A)** Plasma concentrations of MASP1. Box-and-whisker plots show minimum and maximum values, lower and upper quartiles, and median, with each individual value represented as a point superimposed on the graph. Alive (n=47), Dead (n=33). ns-non significant. (**B**) Receiver operating characteristic curves (ROC) show area under curve (AUC) for MASP1.

Supplemental Acknowledgments: Alcohol Hepatitis Network Consortium

Indiana University School of Medicine, Indianapolis, IN: Naga Chalasani, MD; Lauren Nephew, MD; Kavish R. Patidar, DO (currently at Baylor College of Medicine); Raj Vuppalanchi, MD; Niha Samala, MD; Lindsey Yoder, PA; Suthat Liangpunsakul, MD, MPH, Shawna Prange, RN.

Mayo Clinic, Rochester, Minnesota: Vijay H. Shah, MD; Douglas A. Simonetto, MD; Patrick Kamath, MD; Hugo E. Vargas, MD (Mayo Clinic, Scottsdale); Liu Yang, MBBS (Mayo Clinic, Jacksonville)

Cleveland Clinic, Cleveland, Ohio: Srinivasan Dasarathy, MD; Nicole Welch, MD; Annette Bellar; Amy Attaway, MD; Jaividhya Dasarathy, MD (MetroHealth, Cleveland, Ohio); Ashley Growley; David Streem, MD; Laura E. Nagy, PhD

University of Texas Southwestern, Dallas, Texas: Mack C. Mitchell, MD; H. Franklin Herlong, MD; Thomas Kerr, MD; Thomas Cotter, MD

Virginia Commonwealth University, Richmond, Virginia: Arun Sanyal, MBBS; Sara O'Connor, RN; Velimir Luketic, MD; Amon Asgharpour, MD; Stephanie Taylor, RN

University of Louisville, Louisville, Kentucky: Craig J. McClain, MD; Vatsalya Vatsalya, MD, PhD; Loretta Jophlin, MD, PhD; Matt Cave, MD; Suman Kumar Jha, MD; Luis Marsano, MD; Ashutosh Barve, MD, PhD; Jane Frimodig, PhD

University of Pittsburgh, Pittsburgh, Pennsylvania: Ramon Bataller, MD (currently at Universitat de Barcelona, Barcelona, Spain) Samhita Ravi; Jaideep Behari, MD; Sharvari Shivanekar, MD; Paula Novelli, MD; Andres Duarte-Rojo, MD (currently at Northwestern University, Chicago, IL), Naudia Jonassaint, MD

Beth Israel Deaconess Medical Center, Boston, Massachusetts: Gyongyi Szabo, MD, PhD; Michael Curry, MD; Zhenghui G Jiang, MD; Ushma Agarwal; Hannah Siegenberg, Mia Hazel

University of California at San Diego: Bernd Schnabl, MD
NIAAA Project Scientist: Svetlana Radaeva, PhD
