# Supplementary material for: Proteomics identifies complement protein signatures in patients with alcohol-associated hepatitis
Source: JCI Insight. 2024 May 8;9(9):e174127. doi: 10.1172/jci.insight.174127 (PMC11141929; doi:10.1172/jci.insight.174127)
Supplement: Unedited blot and gel images [file jciinsight-9-174127-s013.pdf]

Figure 2 Liver Explants

CD59
#725, CST 65055S, 18kDa
Primary = 1:1000
Secondary = 1:20,000 rabbit
Blocked in 5% MILK + TBST

1m exposure

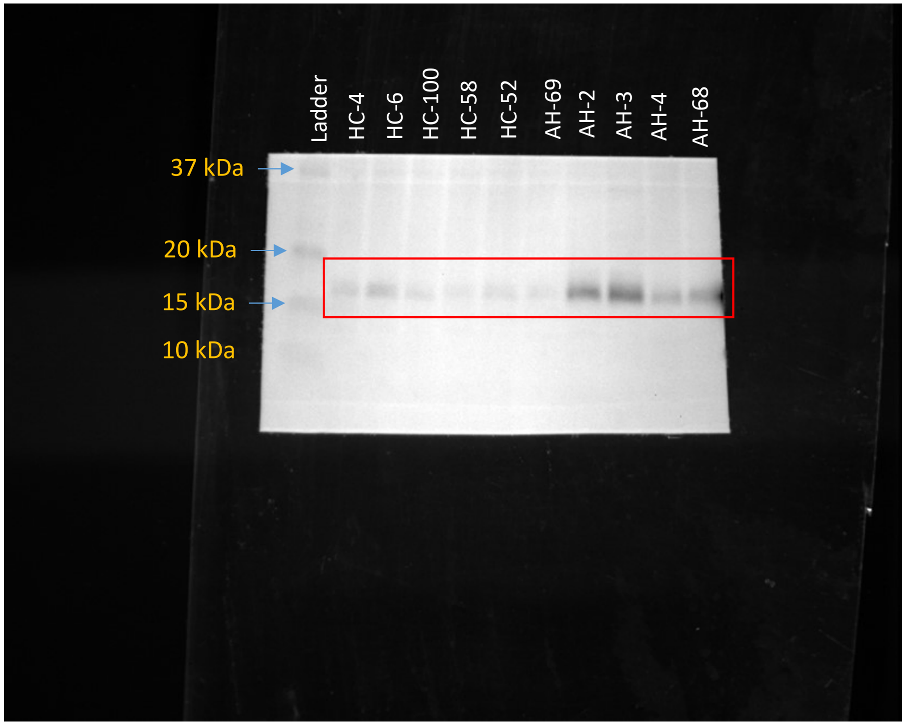

HSC70
#104, SC-7298 santa cruz ~70kDa
Primary = 1:16,000 1hr RT
Secondary = 1:20,000 mouse

1m exposure

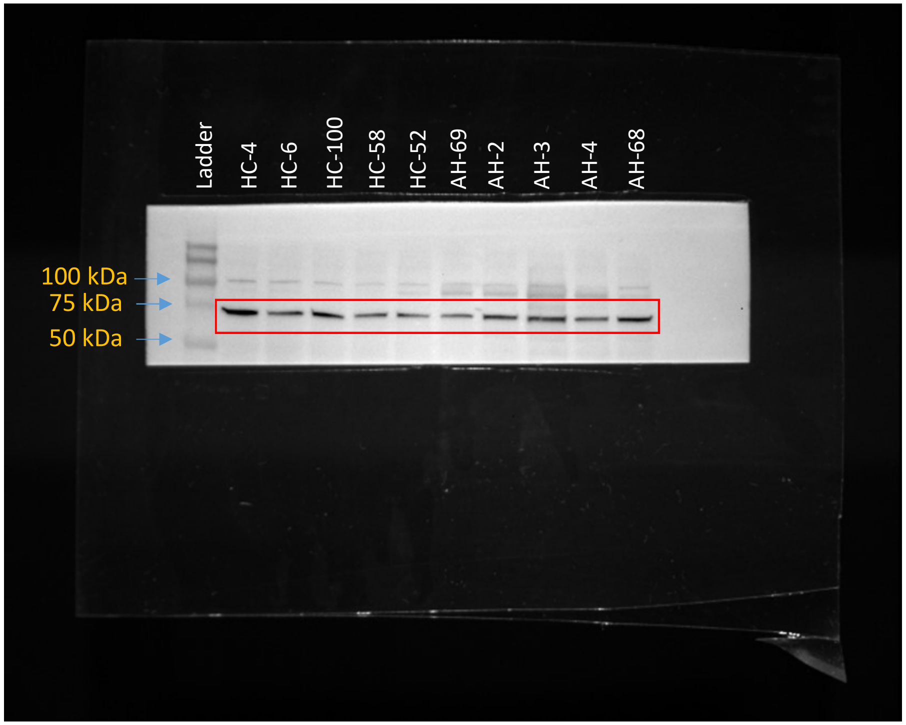

CD59
#725, CST 65055S, 18kDa
Primary = 1:1000
Secondary = 1:20,000 rabbit
Blocked in 5% MILK + TBST

1m exposure

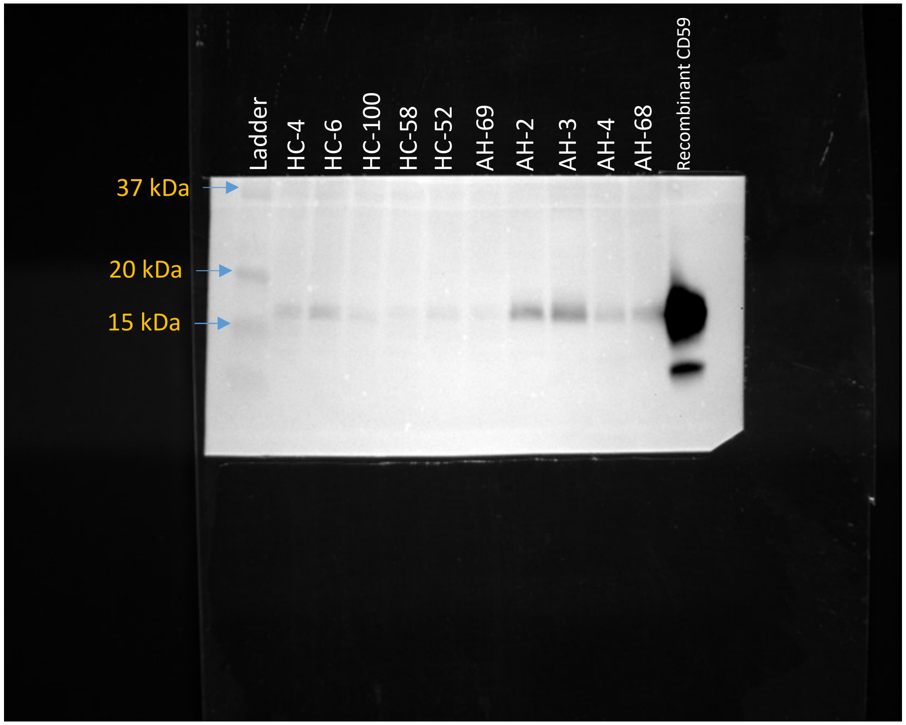

Recombinant protein was
Too strong, cut off
Last lane to re-probe
