# Supplementary material for: Proteomics identifies complement protein signatures in patients with alcohol-associated hepatitis
Source: JCI Insight. 2024 May 8;9(9):e174127. doi: 10.1172/jci.insight.174127 (PMC11141929; doi:10.1172/jci.insight.174127)
Supplement: Supplemental table 14 [file jciinsight-9-174127-s014.pdf]

Supplemental Table 14. Spearman's correlation analysis of complement proteins and pro-inflammatory cytokines in sAH: Test cohort 3

	Pro-inflammatory cytokines																
**Complement Proteins**	**IL33**	**CXCL1**	**IL17E**	**TSLP**	**S100B**	**IFNα**	**IL12**	**IL18**	**TNFα**	**IL8**	**IL17C**	**IL1β**	**IL1α**	**IFNγ**	**IL17**	**CCL20**	**CX3CL1**
**C7**	-0.37	**-0.78**	-0.49	**-0.68**	-0.15	**-0.86**	**-0.67**	**-0.72**	-0.18	-0.40	-0.27	-0.41	-0.33	-0.37	-0.42	-0.26	-0.40
**VSIG4**	-0.22	-0.13	-0.19	-0.09	-0.07	-0.12	-0.03	0.13	0.20	0.23	0.25	0.29	0.25	0.15	0.47	0.40	0.21
**F2**	0.02	0.02	0.19	-0.22	0.41	0.10	0.04	-0.33	-0.33	-0.49	-0.63	-0.39	**-0.66**	-0.24	-0.59	-0.44	-0.47
**COLEC11**	0.27	-0.23	-0.52	-0.21	-0.30	0.09	-0.57	-0.03	0.39	0.18	0.01	0.14	0.25	0.06	0.23	0.27	**0.70**
**CD59**	0.29	0.26	-0.02	0.14	-0.13	**0.64**	-0.10	0.33	0.21	0.18	-0.22	0.14	0.15	-0.13	0.07	0.23	**0.68**
**VTN**	0.00	-0.22	-0.09	-0.26	0.11	0.03	-0.26	-0.08	-0.34	-0.22	-0.29	-0.13	-0.48	-0.34	-0.17	-0.21	-0.40
**C6**	-0.48	-0.58	-0.20	-0.50	0.03	-0.54	-0.17	-0.43	-0.45	-0.33	-0.12	-0.24	-0.43	-0.26	-0.02	-0.21	-0.62
**CFI**	-0.31	-0.26	-0.41	-0.30	-0.03	**-0.68**	-0.53	-0.09	0.48	0.27	0.09	0.26	0.20	0.21	-0.15	0.36	-0.15
**C4BPA**	0.46	**0.70**	0.52	0.56	0.43	0.51	0.33	0.38	0.31	0.28	-0.07	0.24	0.19	0.24	-0.14	0.18	0.34
**FCN2**	**0.79**	0.58	0.59	0.61	0.36	**0.71**	0.26	0.27	-0.01	-0.01	-0.14	-0.03	0.01	-0.18	-0.16	-0.12	0.47
**SERPING1**	0.02	0.22	0.41	0.26	0.63	-0.17	0.12	0.12	0.24	0.36	0.04	0.34	0.20	0.15	0.11	0.47	0.13
**C8A**	0.10	0.11	-0.38	0.01	-0.19	-0.01	-0.46	0.39	**0.66**	0.44	0.16	0.46	0.37	0.21	0.03	0.47	0.31
**COLEC10**	0.21	0.49	0.18	0.34	-0.01	0.37	-0.05	0.36	0.47	0.40	-0.18	0.22	0.39	0.04	-0.24	0.35	0.62
**CFHR5**	-0.09	0.37	0.09	0.17	0.19	0.25	0.42	0.39	0.25	0.37	0.28	0.46	0.17	**0.67**	0.45	0.33	-0.08
**C8G**	0.07	-0.13	-0.28	-0.18	0.19	-0.18	-0.41	0.14	0.23	0.29	0.10	0.37	-0.06	0.25	0.14	0.28	-0.22
**CD93**	-0.18	0.02	0.10	0.30	-0.11	-0.26	0.30	0.25	0.14	0.43	**0.70**	0.36	0.57	0.31	**0.65**	0.40	0.16
**C8B**	0.45	0.37	0.00	0.19	0.22	0.41	-0.15	0.41	0.35	0.30	-0.02	0.36	0.02	0.28	-0.02	0.20	0.12
**FCN3**	0.44	0.22	0.22	0.35	0.24	0.21	-0.12	0.25	0.33	0.20	0.05	0.23	0.29	-0.19	0.08	0.33	0.62
**CFHR4**	-0.41	-0.32	**-0.72**	-0.49	-0.63	-0.09	-0.18	-0.04	0.18	-0.09	0.13	0.02	0.05	0.25	0.24	0.01	-0.04
**CPN1**	0.33	0.59	0.47	0.52	0.58	0.32	0.25	0.60	0.28	0.44	0.17	0.53	0.12	0.28	0.11	0.37	-0.07
**C1QA**	-0.49	-0.57	**-0.69**	-0.49	-0.60	**-0.71**	-0.52	-0.30	0.20	0.18	0.22	0.01	0.27	0.20	0.07	0.15	-0.05
**CLU**	0.19	0.09	0.07	0.11	-0.26	0.16	0.00	-0.15	-0.04	-0.26	-0.20	-0.37	0.04	-0.29	-0.39	-0.32	0.29
**CFB**	0.18	0.63	0.46	0.57	0.20	0.25	0.20	0.44	0.46	0.41	-0.01	0.28	0.45	0.04	-0.24	0.37	0.45
**C1QB**	-0.57	-0.56	-0.58	-0.44	-0.44	**-0.83**	-0.46	-0.24	0.17	0.24	0.29	0.10	0.25	0.19	0.12	0.22	-0.22
**CFH**	-0.24	0.54	0.30	0.38	0.19	0.05	0.13	0.58	0.61	0.46	-0.01	0.46	0.44	0.01	-0.19	0.62	0.24
**CPN2**	-0.26	0.39	0.30	0.14	0.35	-0.05	0.12	0.22	0.30	0.04	-0.30	0.12	-0.02	-0.13	-0.52	0.19	-0.20
**C1QC**	-0.43	-0.55	**-0.64**	-0.42	-0.53	**-0.71**	-0.46	-0.24	0.15	0.22	0.35	0.08	0.27	0.26	0.20	0.15	-0.13
**CFHR1**	0.62	0.16	0.10	0.26	0.32	0.41	0.06	0.24	0.13	0.22	0.28	0.33	0.11	0.29	0.58	0.22	0.42

**CFP**	-0.43	**-0.67**	-0.34	-0.45	-0.12	**-0.81**	-0.23	-0.48	-0.26	-0.13	0.26	-0.13	-0.11	0.04	0.20	-0.10	-0.51
**CR1L**	-0.16	-0.45	-0.03	-0.54	0.10	-0.09	-0.11	**-0.72**	**-0.72**	**-0.72**	**-0.70**	**-0.71**	**-0.80**	-0.48	-0.48	**-0.67**	-0.47
**C4BPB**	0.13	0.55	0.50	0.27	0.42	0.56	0.46	0.21	-0.06	-0.16	-0.40	-0.06	-0.26	-0.08	-0.32	-0.12	-0.06
**MASP1**	-0.03	0.01	-0.19	0.06	-0.57	0.24	-0.12	0.14	0.19	0.05	-0.01	-0.06	0.38	-0.29	0.05	0.13	**0.70**
**C4A**	0.43	0.26	0.18	0.09	0.22	**0.66**	0.31	0.02	-0.23	-0.27	-0.20	-0.13	-0.35	0.12	0.07	-0.32	0.03
**C9**	-0.18	-0.19	-0.21	-0.24	0.05	-0.06	0.09	-0.02	-0.13	0.10	0.20	0.18	-0.13	0.41	0.47	0.08	-0.31
**C4B**	0.13	0.49	0.45	0.25	0.44	0.33	0.22	0.13	0.15	0.00	-0.43	0.00	-0.09	-0.07	-0.43	0.06	0.13
**C1R**	-0.27	-0.40	**-0.68**	-0.43	**-0.75**	-0.06	-0.44	-0.15	0.18	0.07	-0.04	-0.07	0.26	-0.02	0.16	0.13	0.51
**C5**	0.22	0.00	0.16	-0.02	0.60	-0.32	-0.34	-0.15	0.15	-0.02	-0.25	0.05	-0.22	-0.12	-0.43	0.05	-0.22
**CFD**	-0.04	-0.18	-0.14	-0.10	0.02	-0.05	0.09	-0.04	-0.01	0.10	0.28	0.17	0.10	0.29	0.56	0.18	0.11
**CTSG**	-0.21	0.15	0.16	0.28	0.15	-0.16	0.23	0.42	0.20	**0.64**	0.53	0.57	0.44	0.42	0.58	0.58	-0.01
**MBL2**	-0.53	-0.19	0.03	-0.09	-0.04	**-0.74**	-0.09	-0.25	0.12	0.07	0.03	-0.07	0.23	-0.12	-0.29	0.15	-0.16
**C1S**	-0.29	-0.17	-0.55	-0.14	-0.63	-0.34	-0.42	0.12	0.57	0.47	0.29	0.28	**0.64**	0.25	0.15	0.46	0.47
**C2**	0.38	**0.75**	0.40	**0.68**	0.08	**0.66**	0.42	**0.70**	0.37	0.61	0.27	0.48	0.51	0.42	0.28	0.40	0.53
**CALR**	0.35	0.49	0.14	0.44	0.34	0.38	0.09	**0.70**	0.63	0.63	0.38	**0.76**	0.46	0.45	0.54	**0.72**	0.49
**CFHR2**	0.38	0.32	-0.06	0.32	0.10	0.36	-0.10	0.58	0.61	**0.65**	0.36	**0.67**	0.52	0.42	0.58	**0.69**	**0.68**
**C3**	-0.12	0.43	0.27	0.15	0.23	0.17	0.04	0.24	0.26	0.05	-0.44	0.06	-0.04	-0.19	-0.59	0.14	-0.01

R coefficients are given. Correlations with $p < 0.05$ are shown in color (blue for positive correlations and red for negative correlations).
